# The mediating role of financial behavior in the relationship between psychological capital and financial wellbeing: evidence from a cross-sectional study in China

**DOI:** 10.3389/fpsyg.2026.1760291

**Published:** 2026-04-14

**Authors:** Yanping Zhang, Leong-Mow Gooi, Jie Niu

**Affiliations:** 1 Graduate School of Business, SEGi University, Petaling Jaya, Selangor, Malaysia; 2 Mechatronics and Automotive Engineering Department, Xuchang Vocational Technical College, Xuchang, Henan, China; 3 School of Marxism, Zhengzhou University, Zhengzhou, Henan, China; 4 Information Engineering Department, Xuchang Vocational Technical College, Xuchang, Henan, China

**Keywords:** China, conservation of resources theory, financial behavior, financial wellbeing, PLS-SEM, psychological capital

## Abstract

In this paper, we study how Psychological Capital (a higher-order construct of hope, self-efficacy, resilience and optimism) affects Financial Wellbeing of working-age adults in China, where Financial Behavior is the mediator. Based on cross-sectional data of 508 valid subjects recruited by Wenjuanxing online platform, we used a two-step Partial Least Squares Structural Equation Modeling (PLS-SEM) in SmartPLS 4, treating Psychological Capital as a second-order notion and weighting the population weighted by national proportions to increase representation. We found that Psychological Capital does indeed influence Financial wellbeing (*β* = 0.563, *p* < 0.001), but also indirectly affects Financial wellbeing via Financial Behavior (*β* = 0.081, *p* < 0.004), with the same mediation pathway as hypothesized. Weighted data revealed similar structure of effects with slight decrease due to the small effective sample size. The model has solid reliability and discriminant validity and good predictive performance (Stone–Geisser’s *Q*^2^_predict > 0.10; Standardized Root Mean Square Residual (SRMR) = 0.028; Normed Fit Index (NFI) = 0.973) and a smoothness across gender, education, and age. We also find that it is consistent across gender/education and age groups. All of these results support Psychological Capital to be a potentially flexible psychological tool that can positively affect financial wellbeing directly and in a more adaptive manner. This suggests that interventions embedding resilience-building, hope-enhancing, and self-efficacy–strengthening components within financial education may help individuals cultivate more secure long-term financial outcomes. By embedding Psychological Capital within a behavioral explanation framework, we complement the model of financial well-being and provide one of the first population-weighted PLS-SEM studies on the relationships between Psychological Capital and Financial Behavior in China.

## 1 Introduction

Financial wellbeing has received more and more interest recently, following the goals of global policy, such as the United Nations 2030 Sustainable Development Goals. Many countries, even highly advanced and rapidly developing countries, have seen significant economic growth, and financial fragility and stress continue to be prevalent (Antoniades et al., 2020). Financial wellbeing involves the ability to meet current and future promises, to preserve financial stability, and make informed decisions to guarantee a better life (CFPB, 2017). More recently, researchers are realizing that financial wellbeing is not only an economic indicator, but a multi-dimensional concept linked to mental health, subjective wellbeing, and personal productivity (Bashir and Qureshi, 2023; Nanda and Banerjee, 2021).

Based on integrated accounts of financial wellbeing, Brüggen et al. (2017) consider financial well–being as the result of interaction of individual traits, context, and behavior. This is useful because it shows behavior as a proximal and actionable path that more distant drivers such as psychological resources can be used to achieve daily money management and subjective financial wellbeing, though much of the analysis has focused on relatively stable traits or socioeconomic conditions that might not be difficult to adapt at scale, restricting the practical relevance of existing models in interventions.

Based on this model, previous studies have found relatively stable personality traits (e.g., conscientiousness or saving orientations) to lead to financial results (Dare et al., 2023; Riitsalu and Murakas, 2019), but such trait-like traits may not be easily adaptable at scale and they could not be widely adopted in large-scale interventions. Recent results have shown that relatively stable personality traits such as conscientiousness or saving orientations determine financial results (Dare et al., 2023; Riitsalu and Murakas, 2019), while trait-like dispositions may be persistent and/or not easily changed, making them less acceptable for large scale intervention. This has led to a gradual shift in the literature towards state-like psychological resources that, unlike fixed traits, are more flexible, and can be strengthened by intentional focused development (Luthans et al., 2015).

Some other aspects of psychological capital have come up with increasing attention. Whereas psychological capital is often considered a positive psychological state (i.e., hopeful, self-efficacy, resilient, optimistic), psychological capital may be used to help individuals to function in many different aspects (i.e., for emotional control, motivation, performance, etc.) (Avey et al., 2010; Luthans and Youssef-Morgan, 2017; Siu et al., 2014). For financial situations, people with more psychological capital tend to take more conservative financial decisions (e.g., avoid unnecessary debt, save regularly and plan to get ahead of the curve; Goyal et al., 2021; Kim et al., 2019), but the relationship with financial wellbeing has limited empirical evidence, especially in non-western situations, where cultural and structural factors may play an important but incomplete role.

It is also getting renewed attention to the role of financial behavior. As some authors argue, financial behavior provides a very close and immediate driver of financial wellbeing (Chavali et al., 2021) with an accompanying cognitive and emotional resource such as psychological capital (Jumena et al., 2022). According to this interpretation, it is possible to envision psychological or behavioral interventions that promote better financial behavior by exploiting the underlying resource. This mechanism might be particularly relevant in China, where rapid economic growth has not always delivered a result in financial literacy, financial activities and/or financial security (Fan and Lei, 2023).

This is where Psychological Capital, a flexible state-like higher level resource containing hope, self-efficacy, resilience, and optimism (Luthans et al., 2007a) can be theoretically grounded and practically useful for improving financial outcomes. At the same time, growing behavioral finance and wellbeing literature believes that financial behavior (budgeting, saving, debt management, planning) is one of the most important and important factors in financial wellbeing and could be a main mechanism for translating psychological resources into financial outcomes (Brüggen et al., 2017; Xiao and Porto, 2017). However, no experiment explicitly testing the full Psychological Capital → Financial Behavior → Financial Wellbeing pathway has been performed in non-Western contexts such as China, where sociocultural conditions or institutional features can alter behavior and subjective wellbeing.

Another interesting place to be concerned with this perspective is in China. Although there has been significant macroeconomic growth, many working-age adults are still facing long-term financial fears, short-term decisions, and financial behavior, which does not always correspond to long-time welfare. These difficulties correspond to typical sociocultural patterns such as thrift, future planning, and strong-age inter-generational obligations (Hofstede, 2013; Yan, 2010). For many middle-aged adults, as “sandwich generation,” the costs of supporting an aging parent while also supporting a children may be substantial (Chen and Zhou, 2022), and at the same time, cultural expectations of face (mianzi) and social comparisons may encourage conspicuous consumption, resulting in financial pressure (She et al., 2022, 2023). Together, these dynamics suggest that psychological resources may be either buffers or enablers, supporting people to cope with strong financial burdens, and consequently to improve their financial wellbeing.

Against this backdrop, we present a practical investigation of a mediated model where psychological capital serves as a precursor to Financial Behavior as well as better financial wellbeing. As an upper-order construct (Avey et al., 2010; Luthans et al., 2007a), Psychological Capital can be both theoretically intuitive and, in practice, relatively easy to develop, which is of particular interest for financial results. Incorporating psychological capital in the framework described by Brüggen et al. (2017) allows us to study financial wellbeing from a more psychologically tractable perspective rather than purely economic or structural reasons.

Accordingly, this study addresses a clear research gap by testing a theoretically integrated mechanism in China: whether Psychological Capital predicts Financial Wellbeing directly and indirectly through Financial Behavior. We contribute to the literature in three ways. First, we extend Brüggen et al.'s (2017) integrative model by positioning Psychological Capital as a developable psychological antecedent and by explicitly modeling the behavioral transmission channel. Second, we provide context-sensitive evidence from working-age adults in China, where family obligations, social comparison pressures, and rapid socioeconomic changes may amplify the importance of psychological resources for financial adaptation. Third, methodologically, we model Psychological Capital as a formative higher-order construct and evaluate robustness using both unweighted and population-weighted PLS-SEM. Based on Conservation of Resources theory (Hobfoll et al., 2018), we hypothesize that Psychological Capital is positively associated with Financial Wellbeing (H1) and that Financial Behavior mediates this relationship (H2).

The paper is organized as follows: In Section 2, we review the theory of Financial Wellbeing, Psychological Capital and the role of Financial Behavior in a specific context, especially, the understudied Chinese context, and derive our research hypotheses. Section 3 reports the research methodology, including the sampling strategy, data collection procedures, conceptual model specification, measurement instruments for key constructs, and the population weighting method. In Section 4, we report the results including descriptive statistics, measurement model validation (reliability, convergent validity, discriminant validity), structural model testing (direct and indirect effect), and robustness checks (extreme-weight sensitivity analysis, endogeneity tests). In Section 5, the main results, their theoretical contributions to Conservation of Resources Theory and Self-Determination Theory, and their practical implications for financial education, workplace, and fintech platforms in China. In section 6, we summarize the general remarks, acknowledge the limitations of the cross-sectional design and sampling, and suggest directions for future work.

## 2 Literature review

### 2.1 Financial wellbeing

Financial wellbeing refers to the fact that a person has enough resources to meet current and future debts, feel relatively secure about what will come and to make choices that are helpful for a life of satisfaction (CFPB, 2017). Over time, researchers have made a point that financial wellbeing has a multidimensional nature, and that it involves not only objective financial indicators, but also subjective experience of control, perceived security, and satisfaction (Netemeyer et al., 2018). More generally, financial wellbeing measures a person’s ability to bear routine expenses, withstand unexpected shocks and is confident in its long-term financial outlook (Brüggen et al., 2017; Drever et al., 2015; Mahdzan et al., 2019).

As viewed by Sustainable Development Goal 3, financial wellbeing also helps poverty reduction and personal welfare, pointed out in a recent work (Sabri et al., 2023). From this, financial wellbeing is important not only for people to live in the daily budget, but also for societies that strive for economic and social security. This has thus become a topic of a major concern in recent discussions in economics and psychology.

Financial wellbeing can also be measured by objective or subjective measures depending on the model used by the researcher (Brüggen et al., 2017; Netemeyer et al., 2018). Objective measures can take from the concrete information such as income, asset holdings, debt/income ratio, net worth or short term liquidity (Joo and Grable, 2004; Shim et al., 2009). In contrast subjective measures reflect how people see their financial resources, their security, and their ability to meet ongoing financial commitments. These perceptual measures are useful when trying to understand people’s financial life (CFPB, 2017; Xiao and Porto, 2017).

Subjective analysis has become increasingly popular in recent years, partly because it captures psychological and behavioral aspects of objective income-based indicators, especially in settings where higher income does not imply higher financial comfort or stability (Riitsalu and Murakas, 2019). The widely used scale from (CFPB, 2017) reflects the change in sentiment, focusing on people’s self-awareness regarding four aspects of financial life: everyday money management, the freedom of facing financial shocks, perceived progress toward personal financial goals and a freedom to make decisions that improve quality of life. Together, it supports, as it is commonly observed in literature, financial wellbeing involves more than measurable economic resources, including important meanings of control, security and financial confidence (Riitsalu and Murakas, 2019; Xiao and Porto, 2017).

There are a wide range of factors that have shaped financial wellbeing, from objective conditions such as income or debt loads to psychological habits and daily behaviors (Brüggen et al., 2017; Netemeyer et al., 2018). In particular, Financial Behavior has been widely believed as an important factor for financial wellbeing, especially budgeting, saving, debt management, and long-term planning (Dew and Xiao, 2011; Xiao and Porto, 2017). Previous studies have shown that when people struggle with such behaviors, they are frequently subject to stress, are less capable of coping with economic shocks, and finally to lower financial wellbeing (Goyal et al., 2021; Kim et al., 2019). Those who take a steady proactive financial behavior, however, are more stable and more satisfied in financial lives even if they do not have much income (Riitsalu and Murakas, 2019). These findings support the influence of financial behaviors in translating financial resources or psychological properties into actual financial outcomes.

While Financial Behavior is a widely accepted determinant of Financial Wellbeing, more recent researchers are now paying more attention to the internal psychological traits that drive such behavior. In recent research, psychological qualities such as hope, optimism, resilience, and self-efficacy have been shown to affect people’s ability to make financial decisions, and maintain healthy financial habits, particularly in some scenarios, such as in the case of periods of economic uncertainty (Avey et al., 2010; Luthans et al., 2015). This coincides with the context of Brüggen et al. (2017), which has been seen as a result of personal characteristics, behaviors, and external conditions. Under that context, psychological traits are the driving factors that determine how people perceive financial information, react to financial stresses, and make decisions that gradually shape their financial behaviors.

From this perspective, one might expect people having higher Psychological Capital to engage in more proactive and adaptive financial behavior, improve their financial control and improve their wellbeing (Aren and Nayman Hamamci, 2024; Seibert et al., 2024). Together, these considerations give a coherent theoretical basis for viewing Financial Behavior as a bridge between psychological resources and financial wellbeing.

Taken together, existing frameworks and evidence suggest that financial behavior is a proximal driver of financial wellbeing. However, what remains less clear is which developable psychological resources reliably foster such behaviors in ways that translate into improved wellbeing, especially in non-Western contexts where cultural expectations and institutional conditions may alter the psychology–behavior–wellbeing link. This motivates closer attention to Psychological Capital as a state-like resource and to its potential role in shaping financial behavior and financial wellbeing.

### 2.2 Financial wellbeing in China

Financial wellbeing of China stems from a very different nature of culture, psychology and conditions from traditional Western societies. Due to Confucian collectivism, many Chinese individuals find financial satisfaction through meeting their family obligations, being expected across generations and social harmony, rather than solely self-reliant (Chu et al., 2017; Nanda and Banerjee, 2021). On the other hand, Chu et al. (2017) found that a poor internal control, with strong materialistic attitudes, would reduce subjective financial wellbeing, reflecting culturally biased beliefs.

At the same time, financial state of urban China may be influenced by relative income relative to absolute levels. Increasing social competition and the pressure of conspicuous consumption have also, in some cases, increased financial stress (Asadullah et al., 2018). The structure of the situation also complicates things. For example, hukou still has significant rural–urban gap in the accessibility to financial services. This may result in poor experiences of financial wellbeing in different populations (Asadullah et al., 2018).

Although these cultural and structural aspects are important, research on financial wellbeing in China remains limited across psychological and behavioral areas and few studies attempt to link psychological resources, financial behavior and financial wellbeing. In this paper, we attempt to fill this gap by considering how internal psychological resources interact with financial activities to determine financial wellbeing, making use of old ideas and applying them to the unique cultural and institutional context of China.

Recently studies have revealed that psychological capital and perceived empowerment lead to financial wellbeing in different Chinese contexts. Xie et al. (2020) found that financial self-efficacy between vocational students, a crucial component of Psychological Capital, predicts savings and budgeting, and ultimately, subjective financial wellbeing improvement even after considering income and financial knowledge. To et al. (2020) reported that institutional trust and emotional resilience can mask the negative effects of job insecurity for perceived financial wellbeing, which suggests that psychological resources buffering play a key role.

Gender expectations are more challenging. Rural women obtain financial support from their families in financial decisions that they are more autonomous and confident, despite patriarchal constraints (Wang et al., 2025). While China’s rapid growth of digital financial platforms like Alipay and WeChat Pay opens up access to many urban users in many cities, it also opens digital and psychological barriers, especially older adults and less educated rural populations, who may not understand platform literacy or trust well (Lei et al., 2023). This raises the question of financial wellbeing frameworks that fully include psychological capital given China’s heterogeneous socioeconomic and demographic landscape.

Together, these results motivate the need for models that are sensitive to general problems and that bring together internal psychological resources from external socio-institutionalities. In this sense, the proposed integration framework, by Brüggen et al. (2017), that shows how internal capacities, behavioral processes and policy or institutional support interact is a helpful starting point, although a limited amount of research on this framework in Chinese is available. By placing psychological capital and financial behavior in China’s typical sociocultural environment and policy, this paper helps us provide a more context-aware and global understanding of financial wellbeing.

In summary, the Chinese context highlights why psychological resources and daily financial behaviors may be tightly coupled under socio-institutional constraints. Nevertheless, the literature in China remains fragmented: studies often examine financial wellbeing correlates in isolation (e.g., income, attitudes, or single psychological traits) rather than testing an integrated behavioral mechanism. A systematic examination of the PsyCap → Financial Behavior → Financial Wellbeing pathway among working-age adults in China therefore remains an important empirical gap.

### 2.3 Psychological capital and financial wellbeing

Psychological Capital is a higher-level concept of hope, self-efficiency, resilience, and optimism that is increasingly regarded as a valuable personal resource that can affect individuals’ wellbeing and performance (Luthans et al., 2007a; Luthans and Youssef-Morgan, 2017). Psychological capital differs from relatively stable traits, and is able to develop long-term in a fast-changing financial context where people need to adapt to change quickly (Avey et al., 2010). From the viewpoint of Conservation of Resources theory, people with high psychological resources are best placed to protect and mobilize tangible and intangible assets under pressure like financial uncertainty, a fact that is consistent with some recent discussions of how internal resources protect against external stress (Hobfoll et al., 2018).

In each sense, impact of psychological capital may be different, and may interact in an interconnected fashion. For example, self-efficacy may increase people’s faith in facing difficult financial issues such as budgeting, investing or even buying complex products, as reported by Xiao and Porto (2017). Hope helps people to have a plan forward to financial goals, to plan more deliberately and adjust strategy when the situation goes wrong (Barros and Botelho, 2012). Resilience, mentioned by Van Breda (2018), helps people keep financial discipline against job instability, or more serious economic upsets. Optimism contributes to a generally positive financial outlook, and helps people make future-related financial decisions, and usually avoid overly idealistic assumptions (Puri and Robinson, 2007).

Work by other people has added support to these ideas. Liu et al. (2023), for example, reported that Chinese workers with higher psychological capital were more likely to feel more insecure about their financial lives and job satisfaction, which is partially explained by their emotional control and confidence in what the future might be. Seibert et al. (2024) noted that psychological capital not only predicts financial satisfaction directly, but also helps it indirectly through more adaptive cognitive evaluations of personal financial conditions. Studies on students follow this trend, Tian et al. (2020), reported that people with higher psychological capital were less worried about their future financial futures.

In China, where socio-cultural expectations and periodic macroeconomic uncertainty might lead to different psychological motivations, psychological capital may be particularly relevant. Cultural norms that stress familyship, long-term preparation, and harmony may in fact enhance the adaptive potential of high psychological capital and its resiliency and hope factors (Chu et al., 2017). From a perspective of the viewpoint of this picture, the observations relate to the proposed Brüggen et al. (2017) integrative model of financial wellbeing in which psychological traits are fundamental drivers of financial behaviors and individuals’ subjective sense of financial security.

From a theoretical point of view, psychological capital can be understood not only as cognitive and emotional strengths of individuals but also as a motivation that is gained from daily decisions. Conservation of resources Theory clarifies the resource-based logic linking psychological capital with financial wellbeing; a better psychological capital tends to better conserve and deploy resources when under financial strain; Self-Determination Theory clarifying the motivational mechanism in which Financial Behavior is a mediator, in particular, hope and self-efficacy satisfy core psychological needs for competence and autonomy, and support more intentional and self-regulated financial behaviors (Ryan and Deci, 2017). Taking all these aspects in account, there is a two-pathway mechanism, one based on resource protection and one based upon motivational processes, which provides a coherent, convincing theoretical basis to place Psychological Capital as a central predictor of financial wellbeing.

*H1*: Psychological capital is positively associated with financial wellbeing.

This hypothesis draws on old theoretical analysis as well as recent empirical studies. Together, they motivate the growing belief that adaptive psychological resources play a key role in not only individuals’ financial ability but also their long-term satisfaction of their overall economic state.

### 2.4 Mediating role of financial behavior

Financial behavior has become increasingly understood as the way that psychological resources such as psychological capital affect individuals’ financial results. Financial behavior is often understood to be a set of deliberate practices (budgeting, saving, investing, managing debt; Dew and Xiao, 2011; Gutter and Copur, 2011), financial behavior not only maintain day to day financial security, but also shows people’s psychological capabilities. Within Brüggen et al. (2017) context, it is seen as the key link between internal personal traits and financial wellbeing. In other words, it represents the way internal psychological states become tangible financial results, which becomes clearer after considering how people draw on their psychological resources to make financial decisions and problems.

It has been shown that successful and objective financial behaviors predict a level of subjective and objective financial wellbeing, regardless of the income or financial awareness of the individuals (Goyal et al., 2021; Seibert et al., 2024). In some cases, financial behaviors are not only less expensive; they can also be easier to control and lead to a better long term outlook (Dare et al., 2023; Riitsalu and Murakas, 2019). When people spend too much or too much, as may be the case during financial uncertainty, they are often more vulnerable to financial risk and psychological stress (Zainol et al., 2023).

Psychological capital develops behaviors in a different form by increasing individuals’ cognitive, emotional and motivational capacities in ways which eventually become daily behavior. For instance, individuals with higher self-efficacy can be more prepared to take challenging financial choices which in turn induces better budgets and more planning (Xiao and Porto, 2017). Hope fuels the effort to reach financial goals and keeps them active even when the outcome is unknown (Barros and Botelho, 2012). Resilience also helps people cope with financial setbacks, such as temporary unemployment or unexpected costs, and maintain adaptive behaviors (Van Breda, 2018). Optimistic, or at least in the balanced form, is recommended to support forward-looking thinking and strategic risk taking, though over-confidence can become excessive (Aren and Nayman Hamamci, 2024; Puri and Robinson, 2007).

Theoretically, Self-Determination Theory offers a useful framework to analyze this behavioral interaction. It suggests that psychological resources such as self-efficacy and hope fulfill core needs for competence and autonomy (Ryan and Deci, 2017) and after this needs are met, people may be more internally motivated to carry out responsible financial action. To this end, Conservation of Resources theory regards financial behavior as protecting against potential loss of financial resources. With higher Psychological Capital, a higher psychological Capital will benefit from its internal reserves when faced with economic pressure, i.e., adaptive financial behavior is natural to protect against adverse financial consequences (Hobfoll et al., 2018).

Financial behavior has been more and more shown to be a mechanism of connecting psychological capital with financial wellbeing. For example, Kim et al. (2019) found that people of more positive psychological resources take more future-oriented financial practices, which, as their results suggest, helps in financial wellbeing. Further on this line, Seibert et al. (2024) found financial self-regulation and saving behavior to explain their correlation between optimism and financial satisfaction. Bashir and Qureshi (2023) showed hope and resilience that help in financial wellbeing indirectly by supporting more active and deliberate financial management among middle-income workers.

Furthermore, for a bigger Chinese society where institutional trust, risk protection and family-based financial responsibilities have an influence on everyday financial decisions, behavioral mechanisms will also play a greater role (To et al., 2020; Wang et al., 2025). In this setting, it is reasonable to conclude that financial behaviors based on psychological capital may affect not only how people build and manage their own funds, but also how they perceive their ability to meet the expectations and obligations in family and social life.

In other words, Financial Behavior should be not a direct consequence of Psychological Capital, but instead a learning process that relates internal psychological traits in financial behavior. Based on this integrated perspective, we propose the following hypothesis:

*H2*: Financial behavior mediates the relationship between psychological capital and financial wellbeing.

This is similar to a recent hypothesis that psychological characteristics can influence financial wellbeing in most ways by behavior, e.g., it is behaviors that carry the most weight of these psychological factors which motivates the intervention for both psychological and behavioral aspects at the same time (Goyal et al., 2021; Kim et al., 2019; Seibert et al., 2024).

### 2.5 Research gap and hypothesis development summary

Prior work establishes financial behavior as a proximal determinant of financial wellbeing and suggests that positive psychological resources may support adaptive financial decision-making. However, there is limited evidence, especially in China, that simultaneously (a) models Psychological Capital as a higher-order, developable resource, and (b) tests whether it translates into better financial wellbeing through financial behavior. Addressing this gap, we propose and test a mediation framework (PsyCap → FB → FWB) grounded in Conservation of Resources theory. Accordingly, we hypothesize a positive association between Psychological Capital and Financial wellbeing (H1) and a mediating role of Financial Behavior (H2).

## 3 Methods

We received ethical approval from SEGi Research Ethics Committee (SREC), Malaysia (Ethics Approval Number: SEGiEC/SR/GSB/474/2024–2025; approval date: 24 December 2024) in 12 months from December 2024 to November 2025 and the data was collected from January to February 2025.

All human participant forms followed institutional and national guidelines. Participants were required to participate, their answers would not be public, and only for academic purposes. Participants could not stay at any time without consequences, in this case completing and submitting the questionnaire was considered informed consent.

### 3.1 Sample

In this paper, we conducted a cross-sectional quantitative study and recruited working-age adults in mainland China through an online survey. Specifically, we recruited 20–59 years old, who are not only economically active, but also often responsible for household financial activities such as budgeting, saving, children education, retirement planning etc.(Lusardi and Mitchell, 2014; Xiao and Porto, 2017).

A convenience sampling protocol is performed with Wenjuanxing^1^, an online survey website used in China often used in large-scale experiments (Su et al., 2024; Yao et al., 2023). Participants were able to sample in a wide range of regions and socioeconomicities, particularly considering the focus on working-age adults. Before launching the full survey, a pilot test was conducted to check the clarity of items and the reliability of the instrument. No substantive item modifications were made following the pilot; the pilot primarily served to confirm clarity and optimize online survey administration. In total, 528 answers were drawn, and after removing 20 other individuals that fit the age range, 508 valid observations were left.

The sample size was evaluated for adequacy with respect to well-known structural equation modeling guidelines. With 508 valid answers and a recommended minimum of 100 in stable multivariate estimation (Hair, 2010). Previous research indicates that samples of 200–500 are usually acceptable for more complex factor structure models and some authors argue that 500–500 samples can be the best choice for precision or model stability (Mundfrom et al., 2005). Given these two baselines, this sample is within the recommended range and a reliable estimate for PLS-SEM.

However, when population weights were introduced to better representativeness across gender, age, education, and region, the effective sample size ( $n_{eff}$ ) decreased compared to the nominal sample size. According to Kish (1992) standard formulation:


$$n_{eff}=\frac{{\left(\sum_{i=1}^{n}\omega_{i}\right)}^{2}}{\sum_{i=1}^{n}\omega_{i}^{2}}$$

the effective sample size was 130.67, which corresponds to a substantial loss of statistical efficiency relative to the nominal sample size (*N* = 508).

We performed convenience sampling on Wenjuanxing Web platform: the sample may be biased towards urban users that have internet access and higher education levels (similar to the unweighted samples in Table 1 where 65.9% of respondents had college/undergraduate degrees and 53.4% are from eastern China). These could restrict the applicability of the unbound samples to the entire working-age population in China. To reduce bias, and support the outside argument, we further used the population weighting calibrated to the national population margins of age, gender, education and region of Chinese working-aged adults and compared the unweighted and weighted results.

**Table 1:** Demographic characteristics of samples.

(A) Unweighted demographic characteristics			
Variable	Category	*N*	Percentage
Age	20–29	105	20.7%
	30–39	185	36.4%
	40–49	154	30.3%
	50–59	62	12.6%
Gender	Male	163	32.1%
	Female	345	67.9%
Income (RMB/month)	≤3,500	148	29.1%
	3,501–10,000	303	59.6%
	>10,000	57	11.2%
Education	Below college	137	27.0%
	College/Undergraduate	335	65.9%
	Master’s degree	29	5.7%
	Doctorate	7	1.4%

(B) Comparison of unweighted and weighted sample distributions				
Variable	Category	Unweighted_n	Unweighted_%	Weighted_%
Age	20–29	105	20.7	21.07
	30–39	185	36.4	28.19
	40–49	154	30.3	25.58
	50–59	62	12.6	25.16
Gender	Male	163	32.1	51.30
	Female	345	67.9	48.70
Education	Below College	137	27.0	82.29
	College/Undergraduate	335	65.9	16.88
	Master and Doctorate*	36	7.1	0.83
Region	North East	56	11.0	7.00
	East	271	53.4	41.75
	Center	120	23.6	25.70
	West	61	12.0	25.55

(C) Weight diagnostics									
*N*	Min	Max	Mean	SD	P5	P50	P95	P99	CV
508	0.04	9.17	1.00	1.70	0.07	0.26	4.40	9.16	1.70

Panel A reports the unweighted demographic characteristics of the sample (*N* = 508). Panel B compares the unweighted and weighted distributions across demographic categories. Weighted percentages were calculated using normalized probability weights derived from iterative proportional fitting (raking). Panel C reports weight diagnostics, including minimum (Min), maximum (Max), mean, standard deviation (SD), selected percentiles (P5, P50, P95, P99), and the coefficient of variation (CV). * Due to the granularity of national statistical data and the observed sample distribution, master’s and doctoral degrees were combined in the weighting procedure.

This reduction reflects the dispersion of case weights (design effect) and has important inferential implications: standard errors may increase, confidence intervals may widen, and borderline structural effects, particularly indirect effects, may fail to reach conventional significance thresholds under weighting. Accordingly, we treat the population-weighted model as a robustness check for external validity rather than as a direct substitute for the unweighted estimation, and we report results from both specifications throughout the Results section to make this trade-off transparent (Table 2C). To further assess whether the weighted results are driven by extreme weights, we provide weight diagnostics (Table 1C) and conduct an extreme-weight sensitivity analysis using 95th-percentile winsorization (Section 4.6.3; Appendix Table 3). Overall, these analyses show that the core direct effects remain stable under weighting, whereas the mediation pathway becomes more conservative, consistent with the expected consequences of reduced effective sample size and increased heterogeneity under population calibration.

**Table 2:** Structural model results (unweighted and weighted estimations).

(A) Structural results (unweighted model)					
Type	Path	Coefficients	*p*	CI	
				2.5%	97.5%
Direct	Psychological capital - > Financial wellbeing	0.563	0.000	0.488	0.641
	Psychological capital - > Financial behavior	0.469	0.000	0.397	0.546
	Financial behavior - > Financial wellbeing	0.174	0.000	0.085	0.258
Indirect	Psychological capital - > Financial behavior - > Financial wellbeing	0.081	0.000	0.040	0.125
Control variables	CPI- > Financial behavior	0.023	0.600	−0.063	0.107
	CPI - > Financial wellbeing	0.020	0.580	−0.050	0.089
	Disposable income - > Financial behavior	−0.048	0.299	−0.138	0.043
	Disposable income - > Financial wellbeing	0.050	0.201	−0.026	0.128
	Housing price index - > Financial behavior	−0.023	0.591	−0.108	0.063
	Housing price index - > Financial wellbeing	−0.022	0.530	−0.093	0.047

(B) Structural results (population-weighted model)					
Type	Path	Coefficients	*p*	*CI*	
				2.5%	97.5%
Direct	Psychological capital - > Financial wellbeing	0.657	0.000	0.536	0.781
	Psychological capital - > Financial behavior	0.551	0.000	0.416	0.679
	Financial behavior - > Financial wellbeing	0.103	0.141	−0.038	0.237
Indirect	Psychological capital - > Financial behavior - > Financial wellbeing	0.057	0.154	−0.022	0.136
Control variables	CPI- > Financial behavior	0.104	0.134	−0.038	0.238
	CPI - > Financial wellbeing	0.076	0.149	−0.031	0.177
	Disposable income - > Financial behavior	−0.084	0.263	−0.230	0.065
	Disposable income - > Financial wellbeing	0.100	0.130	−0.031	0.228
	Housing price index - > Financial behavior	0.024	0.690	−0.096	0.137
	Housing price index - > Financial wellbeing	−0.084	0.180	−0.203	0.039

(C) Side-by-side comparison of key structural paths						
Type	Path	Unweighted model		Weighted model		*Δβ*
		*β*	*p*	*β*	*p*	
Direct	Psychological capital - > Financial wellbeing	0.563	0.000	0.657	0.000	0.094
	Psychological capital - > Financial behavior	0.469	0.000	0.551	0.000	0.082
	Financial behavior - > Financial wellbeing	0.174	0.000	0.103	0.141	−0.071
Indirect	Psychological capital - > Financial behavior - > Financial wellbeing	0.081	0.000	0.057	0.154	−0.024

Despite this change the last weighted sample still provides enough power and is relatively represented. It also falls in good agreement with well-known standards of structure equation modeling and factor analysis for working-age adults in China. The implications of this efficiency loss for hypothesis support, especially the attenuation of the behavioral mediation under weighting, are discussed in Section 4.6 (see Table 2) and further evaluated via trimming-based sensitivity analyses (Section 4.6.3; Appendix Table 3).

### 3.2 Data collection

A cross-sectional survey was conducted to answer the research questions. The survey used popular measurement scales (Hair et al., 2022) and each construct was translated into Chinese using translation, back translation, and expert reviews to preserve construct validity and have items that agreed with respondents’ Chinese cultures.

Following the rigorous translation-back translation procedure proposed by Brislin (1970), all English-originated scales were adapted to Mandarin Chinese: (1) Two bilingual researchers (with expertise in finance and psychology) independently translated the original scales into Chinese, and resolved inconsistencies to form a preliminary version; (2) A third bilingual native English researcher back-translated the Chinese version into English without accessing the original scales; (3) The research team compared the back-translated version with the original to ensure semantic accuracy, and revised items for cultural relevance; (4) Asking experts in financial behavior and positive psychology evaluated item clarity and construct validity. This process ensured cross-cultural measurement equivalence (Lonner, 1988), and the pilot test (*n* = 203) further confirmed the translated scales’ comprehensibility in the Chinese sample.

Before launching the full survey, we conducted a pilot study to assess item clarity, response process, and preliminary reliability of the instrument in the Wenjuanxing online setting. A total of 203 working-age adults participated in the pilot. No scale items were substantively modified or removed based on the pilot study; instead, the pilot was used to verify the comprehensibility of the translated items and to refine survey administration features (e.g., question ordering, page layout, and instruction wording) to improve readability and reduce potential respondent burden. The pilot results indicated acceptable internal consistency, supporting the suitability of the instrument for the main data collection (DeVellis, 2017; Hair et al., 2022).

We conducted our main survey using Wenjuanxing, an online platform based on website in China. For this to minimize the chance to missed answers, only full questionnaires were available. The built-in constraint was simple. No missing data were present in the final sample, as the Wenjuanxing questionnaire included mandatory filling constraints for all items (respondents could not submit incomplete questionnaires). Consistent with Tabachnick et al. (2019), multivariate outlier screening via Mahalanobis distance confirmed no extreme cases required exclusion.

In order to further test the suitability for multivariate analysis, Mahalanobis distances in IBM SPSS 25.0 have been estimated to screen for multivariant outliers. As indicated by Tabachnick et al. (2019), the chi-square cutoff for the model degree of freedom has been used. No of the cases reached this value. We do not remove the observations after all validation. After all validations, the final data has 508 complete and methodologically reliable answers.

Combined with the multi-stage validation, including the pilot test, completion and outlier screening, helped to ensure that the final sample is as good as structural equation modeling, and the validity of the study.

### 3.3 Conceptual model

**Figure 1:**
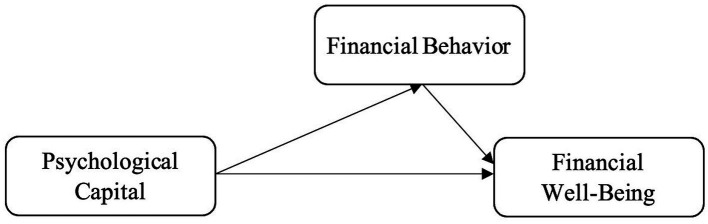
Hypothesized conceptual model. Psychological capital predicts financial wellbeing directly and indirectly via financial behavior.

In this work we propose a theoretically-based conceptual model for financial wellbeing in Chinese adults 20–59 (see Figure 1) and to use financial behavior as a mediator. Based on the financial wellbeing framework (Brüggen et al., 2017), Psychological Capital Theory (Luthans and Youssef-Morgan, 2017), Conservation of Resources Theory (Hobfoll et al., 2018), and Self-Determination Theory (Ryan and Deci, 2017), to incorporate individuals and the nature of psychological resources and behavioral patterns for the understanding of subjective financial wellbeing in China.

As shown in Figure 1, it is assumed that psychological capital influences financial wellbeing in both a direct, and indirect, manner by financial behavior, specifically, financial behavior as the immediate factor of psychological capital and a channel of psychological resources translating to better financial results (Kim et al., 2019; Seibert et al., 2024). This is consistent with recent studies that self-regulating financial actions, budgeting, saving, planning, and others actions play an important role in people’s financial trajectories (Goyal et al., 2021; Xiao and Porto, 2017).

We operationalize this conceptual framework using structural equation modeling, which enables simultaneous estimation of direct and mediated pathways. Considering psychological capital as the ancestor and financial behavior as the mediator, the model offers a more complex picture of how psychological traits influence financial decisions and finally subjective financial wellbeing, thereby providing insight into these dynamics in China’s fast changing socioeconomic landscape and its cultural context, where such relationships can appear in meaningful ways.

### 3.4 Instrument and measurement of variables

The measurement instruments described below were selected to operationalize the focal constructs in the conceptual model (Section 3.3), namely Psychological Capital (as a second-order formative construct), Financial Behavior (mediator), and Financial wellbeing (outcome). This ordering is intended to help readers understand why these constructs matter theoretically before detailing how each construct was measured. A self-based survey was conducted. The survey consisted of four groups of demographic and socio-economic qualities and items of Psychological Capital, Financial Behavior, and Financial wellbeing. All instruments were translated back into Mandarin Chinese according to the procedures (Lonner, 1988). Before the full survey started, 203 participants participated in a pilot test and their experience demonstrated the clarity of the items and that the instrument was always trustworthy.

#### 3.4.1 Financial wellbeing

Financial wellbeing was tested by the 10-item Financial wellbeing Scale developed by the Consumer Financial Protection Bureau (CFPB, 2017). Instead of focusing on feelings or only on objective indicators, the scale explores four aspects of people’s finances: their control over their daily budget, their ability to react to unexpected shocks, their progress toward longer term goals, and their freedom to live in wealth. The subjects are asked how many times each statement applied to them on 5-point Likert (0 = Not at all, 4 = Full) scale.

After a data set, the raw scores were converted from CFPB’s score as a sum index of 0 to 100, where high values are better financial wellbeing. This instrument has the high cross-cultural performance and psychometric validity of many Chinese populations, and has been used in previous studies in Chinese financial behavior, which is further reasons for its applicability in this work (Chavali et al., 2021; Netemeyer et al., 2018).

#### 3.4.2 Psychological capital

Psychological capital was modeled as a second order formative object comprising four reflective first order aspects, self-efficacy, hope, optimism and resilience (Luthans et al., 2007a; Youssef-Morgan and Luthans, 2015). As recommended in higher-order modeling (Hair et al., 2022), the study adopted a two-stage strategy implemented in SmartPLS 4.0. During the first stage, each component of psychological capital was assessed reflectively using established multi-item scales: the 10-item self-efficacy measure developed by Parker (1998), 6-item hope Snyder et al. (1996), 8-item optimism Scheier and Carver (1985), and 10-item resilience Van Der Meer et al. (2018). All items were rated 5-point Likert scale (Strongly Disagree-Strongly Agree).

In the second step latent variable scores over these four dimensions were used as the scaffold of the higher-order psychological capital. By modeling these dimensions as having their own complementary information, psychological capital can be analyzed more closely to affect financial behavior and financial wellbeing (Luthans and Youssef-Morgan, 2017). In order to achieve the quality of our first order constructs, internal consistency with Cronbach’s *α* and Composite Reliability were measured, both above the widely accepted threshold of 0.80.

#### 3.4.3 Financial behavior

Financial Behavior is evaluated based on the 12-item Financial Management Behavior Scale by Dew and Xiao (2011). The scale represents behaviors across five financial contexts: cash management, credit usage, saving and investment, insurance, and retirement. The subjects used 5-point Likert scale (1 = never, 5 = always) to compare their behavior. The scale is chosen not only because of its breadth of theoretical depth, but also because it has a factorial structure and is adapted to cross cultural problems (Dare et al., 2023; Goyal et al., 2021; Riitsalu and Murakas, 2019).

Compared to other commonly used metrics (Atkinson and Messy, 2012), financial management behavior scale is often more reliable and consistent. Prior studies, including in Western and Asian regions, have found that it is consistently successful to capture financial behavior (Xiao and Porto, 2017).

#### 3.4.4 Weighting method description

We weighted the population by precalculated weights before importing the data to SmartPLS to reflect the proportional representation of each respondent according to national distribution of gender, age, education and region given by the National Bureau of Statistics and Wind database. These weights were derived from iteratively proportional fitting (raking) in Stata which slowly expanded the sample to reflect Chinese working-age population (20–59 years). The result better approximated the national population and therefore helped improve the population representation and improved external validity of the estimated models.

The raking weights were treated as sampling (probability) weights rather than frequency weights because they rescale each respondent’s contribution to reflect population margins (age, gender, education, and region) rather than representing case replication. In SmartPLS 4, we specified the weight column as Sampling Weights (Data View → Case Weights → Sampling Weights) and applied it consistently in the PLS algorithm and bootstrapping procedures. All weighted models were estimated using standardized weights with mean = 1.00 (Table 1C). For the sensitivity analysis, we created a P95-winsorized version of the sampling-weight variable (i.e., weights above the 95th percentile were set to P95) and re-estimated the model using the same Sampling Weights setting, differing only in the selected weight column (results see Appendix Table 3).

#### 3.4.5 Control variables

To account for eliminating the impact form regional macroeconomic conditions that may shape individuals’ financial experiences beyond psychological resources, we included three province-level indicators as control variables: consumer price index (CPI), per-capita disposable income, and the housing price index. We included CPI to capture cost-of-living pressure, which is shown to be meaningfully related to subjective assessments of material and financial wellbeing beyond individual characteristics (Asadullah et al., 2018). We additionally controlled for disposable income and housing price conditions because objective economic resources and housing-market dynamics can systematically shape perceived financial wellbeing through income security and wealth/collateral channels (Atalay and Edwards, 2022; Bufe et al., 2022).

These three variables are all sourced from provincial economic development data published on the official website of the China National Bureau of Statistics^2^. Each respondent was matched to these macroeconomic indicators based on their province (or corresponding city-level index where applicable) and the survey period. All control variables were standardized prior to model estimation to facilitate coefficient comparability. In the structural model, the controls were specified as exogenous predictors of Financial Behavior and Financial wellbeing in both unweighted and population-weighted PLS-SEM estimations.

## 4 Findings and discussions

### 4.1 Data analysis

Data were analyzed in SPSS and SmartPLS following a PLS-SEM procedure. We imported the data and added simple data cleaning and descriptive statistics such as minimum and maximum values, mean, standard deviations, in SPSS for further investigation of univariate patterns. We performed analysis in SmartPLS. In total, we checked internal consistency and construct reliability via Cronbach alpha, *ρ*_A and ρ_C; all more than the popular 0.70 threshold (Hair, 2010). We verified convergent validity using AVE values above 0.50 and assessed collinearity using variance inflation factors (VIF), VIF values below 3.0 indicate no serious multicollinearity (Hair et al., 2022).

Using the PLS algorithm the model derived outer loadings, path coefficients, and R^2^ values of the endogenous constructs, and fit was checked through SRMR which adds additional uncertainty in global specification. To test the hypotheses and Financial Behavior, we bootstrap with 10,000 subsamples for more stable inference, especially in complex PLS-SEM models.

### 4.2 Profile of respondents

The profile of respondents is shown in Table 1 below.

Table 1A presents the information of 508 valid respondents. Age: Our sample has been dominated by those age 30–49 (66.7%), in line with the study’s focus on financially active working-age adults. Women ages 20–29 are 20.7% of participants and 50–59 have 12.6% of the subjects. Women (67.9%) is more than men (32.1%). Based on monthly income, 59.6% of respondents reported earning RMB 3501 to 10,000, 29.1% reported below RMB 3500, and 11.2% reported above RMB 10000. For education, 65.9% reported college/undergraduate, 27.0% reported below-college, and 7.1%, respectively, reported postgraduate degree (master or doctorate), reflecting the expanding prevalence of higher education among working-age adults in China.

To increase population representativeness, we used probability weights calibrated to national demographic margins of age, gender, education, region. Table 1B presents a comparison between the weighted and the weighted distributions. After weighting, the gender is roughly balanced (male = 51.30%; female = 48.70%). The weighted age distribution has more focus on 50–49 and is smaller relative for 30–39 and 40–49 than for the unweighted sample. Education is the largest variable: the weighted distribution has a larger share for below- college (82.29%) and less share for college/graduate (16.88%) and postgraduate education (0.83%). Master and Doctorate degrees were combined for the weighted calculation due to the quality of national statistics and the distribution of the sample. Regionally, weights shift the East (41.75%) share of the unbalanced sample and give a larger chunk of the Center (25.70%) and West (25–55%) to the population margins used for calibration.

Finally, Table 1C reports diagnostic statistics for the sampling-weight variable to evaluate the potential influence of extreme weights. The weights have a mean of 1.00 and range from 0.04 to 9.17, with a standard deviation of 1.70 (CV = 1.70). The 95th percentile is 4.40, indicating moderate dispersion and a right-tailed distribution of weights. These diagnostics provide transparency regarding the weighting procedure and allow readers to assess whether a small number of highly weighted cases could disproportionately affect the weighted PLS-SEM estimates.

We therefore conducted a trimming-based sensitivity analysis to evaluate whether the weighted structural results are unduly influenced by extreme case weights (see Section 4.6.3 and Appendix Table 3).

### 4.3 Descriptive statistics

The profile of respondents is shown in Table 3 below.

**Table 3:** Descriptive statistics of variables.

Variables	*N*	Min	Max	Mean (M)	Standard deviation (SD)
Hope	508	1.17	5.00	3.8209	0.91122
Self-efficacy	508	1.30	5.00	3.6909	1.01701
Resilience	508	1.20	4.90	3.7079	0.99370
Optimism	508	1.38	5.00	3.8027	0.92214
Financial behavior	508	1.42	4.75	3.6055	1.04592
Financial wellbeing	508	25.00	81.00	58.7657	13.90863

Table 3 summarizes the statistics of the key study variables, psychological capital, financial behavior, and financial wellbeing. The four measures of Psychological Capital are typically of high importance: hope (*M* = 3.8209, SD = 0.91122), self-efficacy (*M* = 3.6909, SD = 1.01701), resilience (*M* = 3.7079, SD = 0.9937), and optimism (*M* = 3.8027, SD = 0.92214). All the values are five-point Likert. Together, these results suggest that respondents reported relatively high psychological resources, consistent with prior work on PsyCap and perceived control (Goyal et al., 2021; Luthans and Youssef-Morgan, 2017).

The mean score in Financial Behavior was 3.6055 (SD = 1.04592), which indicates that some people prefer to be in a positive financial practice (saving, planning, budgeting, etc.) and the variation in responses suggest that some individuals exhibit behavioral differences, as have been argued by several researchers, that might be related to differences in financial literacy, motivation, or socio-economic level (Seibert et al., 2024; Xiao and Porto, 2017).

Financial wellbeing based on CFPB level value, with mean 58.7657 (SD = 13.90863) indicates that some people feel safe, but the wide range of scores (25–81) shows that people’s own wellbeing is quite different across individuals, probably due to structural differences, psychological factors, and day-to-day financial behavior (Brüggen et al., 2017; Lusardi and Mitchell, 2014). Taken as a whole, this variability raises the question whether structural equation modeling could aid understand how psychological capital relates to financial wellbeing.

### 4.4 Internal consistency and construct reliability

Table 4 summarizes the results of internal consistency and convergent validity checks based on Cronbach alpha, *ρ*_A, *ρ*_C, and AVE. In turn, Table 5 considers discriminant validity using Heterotrait–Monotrait (HTMT) ratio of correlations, which is widely considered to be more sensitive in determining if a particular construct is unique in structural equation modelling.

**Table 4:** Reliability and convergent validity of latent constructs (unweighted and weighted models).

Model	Variables	Cronbach’s *α*	*ρ*_A	*ρ*_C	AVE
Unweighted Model	Hope	0.873	0.874	0.904	0.612
	Self-efficacy	0.940	0.941	0.949	0.650
	Resilience	0.936	0.937	0.946	0.636
	Optimism	0.910	0.911	0.927	0.615
	Financial behavior	0.950	0.950	0.956	0.646
	Financial wellbeing	–	–	–	–
Weighted model	Hope	0.870	0.873	0.902	0.606
	Self-efficacy	0.942	0.943	0.951	0.659
	Resilience	0.935	0.937	0.945	0.633
	Optimism	0.922	0.923	0.936	0.647
	Financial behavior	0.950	0.952	0.956	0.647
	Financial wellbeing	–	–	–	–

Reliability and convergent validity indices are reported for both unweighted and population-weighted estimations. Financial wellbeing was operationalized as the CFPB-transformed score (FWB_Scores) and modeled as a single-indicator construct; therefore, internal consistency metrics (Cronbach’s α, ρ_A, ρ_C, AVE) are not applicable for this construct. Full indicator outer loadings for reflective constructs are reported in Appendix Table 2.

**Table 5:** Discriminant validity of variables (HTMT).

Variables	Hope	Self-efficacy	Resilience	Optimism	Financial behavior	Financial wellbeing
Hope						
Self-efficacy	0.286					
Resilience	0.437	0.492				
Optimism	0.383	0.349	0.425			
Financial behavior	0.372	0.339	0.390	0.365		
Financial wellbeing	0.470	0.469	0.542	0.455	0.449	

The measurement model demonstrated satisfactory psychometric properties in both the unweighted and population-weighted estimations. As shown in Table 4, internal consistency reliability was high across constructs: Cronbach’s *α* and composite reliability indices (*ρ*_A and *ρ*_C) consistently exceeded the recommended threshold of 0.70 in both models (Hair et al., 2022; Nunnally and Bernstein, 1994). Specifically, *ρ*_C ranged from 0.904 to 0.956 in the unweighted model and from 0.902 to 0.956 in the weighted model, indicating strong internal consistency and stable reliability after applying sampling weights.

Convergent validity was also supported. All constructs exhibited AVE values above 0.50 in both estimations (Fornell and Larcker, 1981), suggesting that each latent variable explains more than half of the variance in its indicators. Importantly, the weighted AVE values (Hope = 0.606; Self-Efficacy = 0.659; Resilience = 0.633; Optimism = 0.647; Financial Behavior = 0.647) were highly comparable to their unweighted counterparts, implying that population weighting did not materially alter the measurement quality.

Finally, to address item-level reliability, we examined the outer loadings for all indicators. The results indicate that indicator loadings are acceptable and generally exceed the 0.70 guideline (Hair et al., 2022). For transparency and to comply with full reporting standards for the weighted estimation, the complete outer loading matrices for both the unweighted and weighted models are provided in Appendix Table 2.

Discriminant validity was assessed using the heterotrait–monotrait ratio (HTMT). Following established guidelines, HTMT values below 0.85 indicate strict discriminant validity, while values below 0.90 are often considered acceptable in applied research (Hair et al., 2022; Henseler et al., 2015). As shown in Table 5, all HTMT values are below these thresholds, supporting adequate discriminant validity among the Psychological Capital dimensions and between Psychological Capital dimensions, Financial Behavior, and Financial wellbeing.

All these results support the quality of the measurement model and provide a basis to further structural path analysis. As another verification, Section 4.4.1 also showed the redundancy validity of Psychological Capital and shows that it converges to global I-PCQ criterion.

#### 4.4.1 Redundancy validity of psychological capital

In order to further test the usefulness of the second-order formative construct Psychological Capital, we replicated the relationship to the short Psychological Capital Questionnaire (I-PCQ) a global criteria measure (Hair et al., 2022). Given what is known about a formative higher order construct, it will be expected to have a significant (typically significant and statistically significant) connection to a potentially meaningful global criterion (Henseler et al., 2015) and hence allow us to measure whether the higher order build is adequate for its purpose.

As shown, Psychological Capital predicts the I-PCQ score (*β* = 0.571, *p* < 0.001), which explains 32.6% of its variance (*R*^2^ = 0.326). Table 6 is the full summary. Overall, our results provide clear evidence for redundancy, indicating that the second-order psychological capital does not only reflect the fundamental conceptual setting of psychological capital, but also closely fits a global measure.

**Table 6:** Result of redundancy validity of psychological capital.

Path	*β*	*t*-value	*p*-value	95% CI	*R*^2^ (target)	*N*	Bootstraps
PsyCap → I-PCQ	0.571	16.361	0.000	[0.505, 0.642]	0.326	508	10,000

Taken together, this result lends strong support to the psychometric soundness of the psychological capital measurement specification and, at the same time, reinforces the suitability of using a two-stage procedure to model psychological capital as a formative higher-order construct.

### 4.5 Correlations and collinearity diagnostics

Table 7 reports the Pearson correlation coefficients, offering a straightforward view of the bivariate relationships among the main study variables, including the first-order components of Psychological Capital, Financial Behavior, and Financial wellbeing. As a complementary check, Table 8 summarizes the VIF-based collinearity diagnostics for both stages of the measurement model. Taken together, these results suggest that multicollinearity is not a substantive issue and that the indicators operate independently enough to support the subsequent analyses.

**Table 7:** Pearson correlation coefficients among original variables coming out of the first stage.

Variables	Hope	Self-efficacy	Resilience	Optimism	Financial behavior	Financial wellbeing
Hope	1.000					
Self-efficacy	0.258 (0.000)	1.000				
Resilience	0.395 (0.000)	0.461 (0.000)	1.000			
Optimism	0.342 (0.000)	0.323 (0.000)	0.392 (0.000)	1.000		
Financial Behavior	0.339 (0.000)	0.321 (0.000)	0.367 (0.000)	0.340 (0.000)	1.000	
Financial wellbeing	0.438 (0.000)	0.455 (0.000)	0.525 (0.000)	0.435 (0.000)	0.437 (0.000)	1.000

The value of *p* is in parentheses.

**Table 8:** Collinearity diagnostics for the two-stage measurement model of psychological capital.

Variable relationship	VIF
First stage	
Financial behavior - > Financial wellbeing	1.275
Hope - > Psychological capital	1.252
Optimism - > Psychological capital	1.277
Resilience - > Psychological capital	1.494
Self-efficacy - > Psychological capital	1.319
Second stage	
Financial behavior - > Financial wellbeing	1.289
Psychological capital - > Financial behavior	1.000
Psychological capital - > Financial wellbeing	1.289

To assess the relationships among the core variables, Pearson correlation coefficients were calculated for the four first-order dimensions of Psychological Capital, hope, self-efficacy, resilience, and optimism, alongside Financial Behavior and Financial wellbeing (see Table 7). All dimensions, however, exhibited strong and positive correlations with both Financial Behavior and Financial wellbeing (*p* < 0.001), consistent with theoretical expectations and potentially valid for these groups. Among these, resilience was strongest with Financial wellbeing (*r* = 0.525), as has been found before in its role in mitigating financial stress and adaptive coping (Aren and Nayman Hamamci, 2024; Van Breda, 2018).

Similar relationships were found between the four psychological capital dimensions of interest with coefficients between 0.258 and 0.461. This is not too large and it suggests an empirical explanation of using psychological capital as a single second order unit Luthans and Youssef-Morgan (2017). From this line of thinking, it seems reasonable to consider psychological capital to be a higher order formative in this structure.

In order to further evaluate the possible multicollinearity of predictors, VIFs were calculated for all paths (Table 8). Under our model rules, all VIF values were close to the conservative cut-off (Hair, 2010; Kock and Lynn, 2012). The range between 1.252 and 1.494 is small but suggests that collinearity is unlikely to be a serious concern at each stage of the model. As expected, the single-predictor path from Psychological Capital to Financial Behavior gave a VIF of 1.000, which is typical for models with only one intra-exogenous predictor (Hair et al., 2022).

Altogether, the correlation patterns and collinearity diagnostics demonstrated theoretically and empirically that both measurement and structural components of the model are consistent and are sufficient for the testing of hypothesis following.

#### 4.5.1 Formative weights of second-order PsyCap

Psychical Capital is a formative higher-order framework made up of four first-order dimensions: hope, optimism, resilience, and self-efficacy. We compare these dimension’s contribution from bootstrap-based significance tests and collinearity tests in Appendix Table 1. For unweighted estimation, all four dimensions contribute positively and significantly to Psychological Capital (all *p* < 0.001), the one with the largest weight is resilience (*β* = 0.390), and the others are hope (*β* = 0.350), self-efficiency (*β* = 0.326), and optimism (*β* = 0.312). In weighted estimation, the contribution is broadly similar, which in turn boosts resilience again (*β* = 0.429), optimism (*β* = 0.394), and hope (*β* = 0.325) with all *p* < 0.001. Weight of self-efficacy (*β* = 0.181, *p* = 0.056) is marginally lower, but its confidence interval remains above 0, suggesting a weaker but non-negligible contribution once population representativeness is imposed. VIF values for both estimates are small (approximately 1.25–1.71), multicollinearities between these dimension are unlikely to influence the estimates.

### 4.6 Structural model result and hypothesis testing

In order to estimate the structure model with higher order constructs, we performed the two-stage procedure in partial least squares structural equation modeling (PLS-SEM) (Hair et al., 2022). In the first stage, latent variable scores for psychological capital, hope, self-efficacy, resilience, and optimism were produced, which were subsequently transformed into the second stage to determine the formative second order construct. Based on these inputs, our full structure model is estimated in SmartPLS, which allows us to study relationships between key variables more directly.

**Figure 2:**
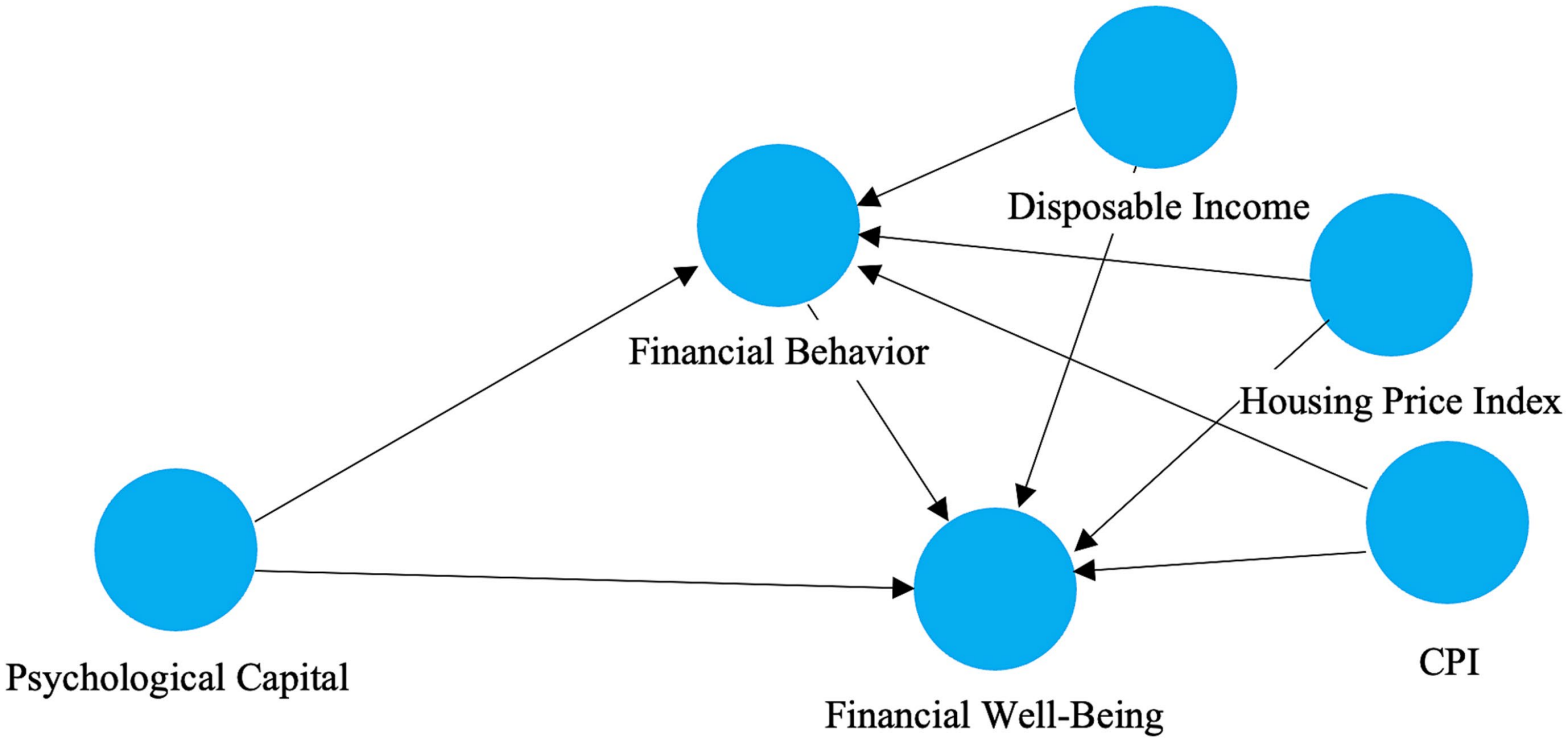
Structural model including macroeconomic control variables. CPI, disposable income, and housing price index are specified as exogenous predictors of financial behavior and financial wellbeing.

In order to account for provincial macroeconomic conditions, we include three external control variables, CPI, disposable income, and housing price index (see Figure 2) in both the unweighted and population weighted second stage PLS-SEM models. These macro-level quantities may impact people’s financial activity and wellbeing via regional cost-of-living pressure, income level and asset prices. Inclusion of these measures, however, decreases the possibility that the estimated psychological trends are biased by external economic conditions.

**Figure 3:**
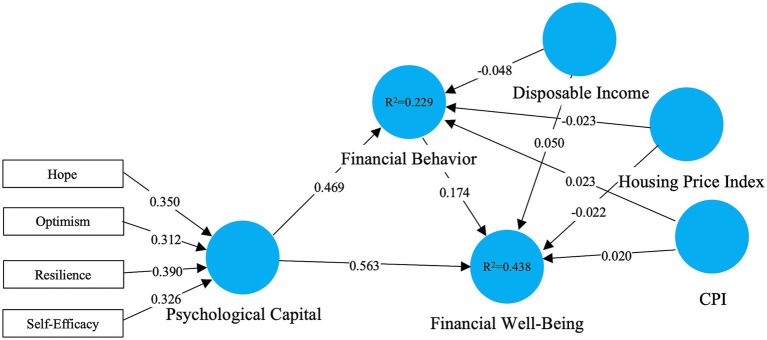
Unweighted PLS-SEM results (second stage). Standardized path coefficients and *R*^2^ values are shown for the unweighted model; formative outer weights for the PsyCap dimensions are displayed on the second-order construct.

In order to assess the relevance of structural paths, we bootstrapped 10,000 resamples, providing steady standard errors and confidence intervals. The unweighted model with control variables results is illustrated in Figure 3 and the direct and indirect effects provided by mediation analysis are shown in Table 2.

Path coefficients and significance were tested based on bootstrapping 10,000 resamples in SmartPLS (Hair et al., 2022). In unweighted model results (Figure 3 and Table 2A), all hypothesized relationships among Psychological Capital, Financial Behavior, and Financial wellbeing were significant (*p* < 0.001), providing strong support for our proposed mechanism.

Psychological Capital showed a sizable and clearly significant direct effect on Financial wellbeing (*β* = 0.563, *p* < 0.001; Table 2A and Figure 3), thereby lending empirical backing to Hypothesis 1 (H1). This pattern echoes earlier work suggesting that individuals drawing on stronger psychological resources, such as hope, resilience, optimism, and self-efficacy, tend to report greater financial satisfaction and reduced stress (Lim et al., 2014; Luthans and Youssef-Morgan, 2017). From this perspective, we might consider the internal psychological resources to play a pivotal role in subjective financial wellbeing.

Besides, Psychological Capital positively predicted Financial Behavior (*β* = 0.469, *p* < 0.001; Table 2A and Figure 3), and Financial Behavior also positively predicted Financial Wellbeing (*β* = 0.174, *p* < 0.001; Table 2A and Figure 3). The indirect effect of Psychological Capital on Financial Wellbeing via Financial Behavior was also significant (*β* = 0.081, *p* < 0.001; Table 2A), suggesting a partial mediation effect and supporting H2. This relationship with Conservation of Resources Theory (Hobfoll, 2010; Hobfoll et al., 2018), which suggests that people who are endowed with more psychological resources are more inclined to adopt adaptive financial behaviors that protect and improve their wellbeing, and is compatible with the behavior described in Brüggen et al. (2017) model of financial wellbeing, in which psychological strengths are translated into financial outcomes through behavioral channels.

Besides the main paths, Table 2A provides the structural effects of macroeconomic controls (CPI, disposable income, housing price index) on financial behavior and financial wellbeing. For all six controls the coefficients are small (|*β*| > 0.050) and statistically significant (all *p* > 0.201) with bootstrap confidence intervals spanning zero. This suggests that, for the sample and modeling specification considered in this sample, provincial-level cost-of-living pressure, income conditions and housing-market conditions do not cause meaningful incremental variance in individual financial behavior or perceived financial wellbeing if psychological capital and financial behavior are considered. Furthermore, without these macroeconomic control, the estimated results indicate that these associations are mainly dependent on individual-level psychological resources and behavioral channels rather than confounding broad regional economic conditions.

In summary, our results confirm both direct and indirect effects of this model and also provide solid evidence for the proposed mechanism.

**Figure 4:**
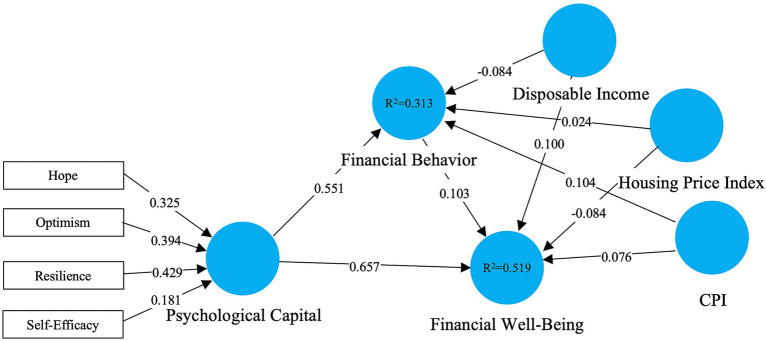
Population-weighted PLS-SEM results (second stage). Standardized path coefficients and *R*^2^ values are shown for the weighted model; formative outer weights for the PsyCap dimensions are displayed on the second-order construct.

In order to improve external accuracy and align the sample with the national population structure, we applied probability weights calibrated to the demographic distributions of age, gender, education, and region, and re-estimated the model using population-weighted PLS-SEM. To facilitate direct comparison across estimation schemes, Table 2C presents a side-by-side summary of all key structural paths for the unweighted and weighted models, including the macroeconomic control paths. The full weighted-model estimates are reported in Table 2B and illustrated in Figure 4.

As shown in Table 2B, the main direct effects of Psychological Capital were stable and significant after weighting. Psychological Capital continues to predict Financial Behavior (weighted *β* = 0.551, *p* < 0.001), and Financial wellbeing (weighting *β* = 0.657, *p* < 0.001), with confidence intervals that do not exceed zero, indicating that they are valid even for population representativeness. By contrast, the behavioral pathway from Financial Behavior to Financial wellbeing weakened and became statistically non-significant in the weighted estimation (weighted *β* = 0.103, *p* = 0.141; Table 2B and Figure 4), and the corresponding indirect effect (Psychological Capital → Financial Behavior → Financial wellbeing) was also non-significant (weighted *β* = 0.057, *p* = 0.154; Table 2B). This pattern suggests that the mediation evidence supporting Hypothesis 2 is not robust once weighting is imposed; accordingly, the mediation should be described as supported in the unweighted (sample-based) model but not conclusively generalizable under the weighted specification.

The non-significant indirect effect in the weighted estimation has several implications for interpretation. First, it indicates that the behavioral transmission mechanism may be more sensitive to demographic composition, meaning that the extent to which financial behavior translates psychological capital into financial wellbeing could vary across segments defined by age, gender, education, and region. Second, although reduced effective sample size under weighting can weaken statistical power, the loss of significance should not be interpreted solely as a power artifact; rather, it warrants a more cautious substantive claim that psychological capital exerts a robust direct association with financial wellbeing, whereas its indirect association via financial behavior may be contingent on demographic heterogeneity captured by the weighting scheme. Accordingly, the weighted model strengthens confidence in the external validity of the direct effects (Table 2C) while highlighting that the mediation pathway requires further confirmation using larger probability-based samples and analyses that explicitly model heterogeneity (e.g., multi-group analysis across age, gender, education, and region).

Table 2B additionally indicates that the effects of the macroeconomic controls (CPI, disposable income, and housing price index) on Financial Behavior and Financial wellbeing remain small in magnitude and statistically non-significant under weighting (*p* ≥ 0.130), with bootstrap confidence intervals spanning zero. Substantively, these results suggest that, once population representativeness is accounted for and the individual-level psychological mechanism is modeled, province-level macroeconomic conditions provide limited incremental explanatory power for individual financial behavior and perceived financial wellbeing in this dataset. The non-significance of the controls also implies that the attenuation of the behavioral pathway after weighting is unlikely to be driven by omitted macroeconomic confounding; instead, it is more plausibly attributable to reduced effective sample size and increased heterogeneity induced by applying population weights (Gelman, 2007; Hair et al., 2022).

In sum, the weighted estimation supports the stability and external validity of the direct effects of Psychological Capital on both Financial Behavior and Financial wellbeing. However, the mediation pathway through Financial Behavior becomes statistically non-significant after weighting, suggesting that the indirect mechanism should be interpreted cautiously and may be more heterogeneous across population subgroups. Accordingly, our strongest conclusion is that Psychological Capital is a robust predictor of financial wellbeing, while the behavioral mediation requires further evidence under probability-based, adequately powered designs.

#### 4.6.1 Predictive power assessment (PLSpredict)

PLSpredict was conducted on both the unweighted and population-weighted datasets. For transparency and readability, we report both unweighted and weighted PLSpredict results in the main text (Table 9). This presentation is consistent with the role of weighting in this study (i.e., improving population representativeness) while keeping the primary predictive diagnostics anchored in the full nominal sample; importantly, the weighted PLSpredict results lead to the same substantive conclusion regarding predictive relevance. For analyzing the out-of-sample prediction performance of PLSpredict using 10-fold cross validation, Hair et al. (2022) and Shmueli et al. (2019). Table 9 shows prediction errors of the PLS-SEM model with respect to a linear regression benchmark (LM) for all indicators of financial behavior and financial wellbeing. In PLSpredict, predictive performance of the PLS-SEM model is benchmarked against a naive linear model (LM). For each indicator, a “PLS win” is recorded when the PLS-SEM prediction error (RMSE and/or MAE) is lower than the corresponding LM error, indicating superior out-of-sample predictive accuracy (Hair et al., 2022; Shmueli et al., 2019). Conversely, when LM errors are lower, the benchmark “wins,” suggesting that the PLS-SEM model does not add predictive value for that indicator. Following prior guidance, we interpret predictive relevance by considering (a) the sign and magnitude of Q^2^_predict (values > 0 indicate predictive relevance), and (b) whether PLS-SEM achieves more wins than losses across the indicators of the endogenous constructs.

**Table 9:** PLSpredict results (unweighted and weighted model).

(A) Unweighted model							
Construct	Indicator	RMSE (PLS)	RMSE (LM)	MAE (PLS)	MAE (LM)	*Q*^2^_predict	Predictive outcome
Financial behavior	Fin_Be01	1.225	1.234	1.002	1.008	0.128	PLS win
	Fin_Be02	1.208	1.216	0.980	0.986	0.131	PLS win
	Fin_Be03	1.224	1.231	0.985	0.989	0.150	PLS win
	Fin_Be04	1.217	1.223	0.990	0.996	0.138	PLS win
	Fin_Be05	1.203	1.211	0.985	0.988	0.137	PLS win
	Fin_Be06	1.222	1.228	0.996	1.001	0.117	PLS win
	Fin_Be07	1.207	1.211	0.988	0.993	0.144	PLS win
	Fin_Be08	1.234	1.242	0.993	1.003	0.111	PLS win
	Fin_Be09	1.192	1.200	0.968	0.972	0.143	PLS win
	Fin_Be10	1.202	1.208	0.971	0.974	0.141	PLS win
	Fin_Be11	1.216	1.223	1.000	1.005	0.140	PLS win
	Fin_Be12	1.195	1.202	0.962	0.965	0.144	PLS win
Financial Wellbeing	FWB_Scores	10.810	10.834	8.052	8.054	0.397	PLS win

(B) Weighted model							
Construct	Indicator	RMSE (PLS)	RMSE (LM)	MAE (PLS)	MAE (LM)	Q^2^_predict	Predictive outcome
Financial behavior	Fin_Be01	1.224	1.234	1.001	1.007	0.129	PLS win
	Fin_Be02	1.208	1.215	0.979	0.986	0.131	PLS win
	Fin_Be03	1.224	1.231	0.985	0.989	0.150	PLS win
	Fin_Be04	1.216	1.222	0.989	0.994	0.140	PLS win
	Fin_Be05	1.203	1.211	0.986	0.989	0.137	PLS win
	Fin_Be06	1.222	1.228	0.996	1.001	0.117	PLS win
	Fin_Be07	1.206	1.211	0.987	0.993	0.144	PLS win
	Fin_Be08	1.233	1.241	0.992	1.002	0.112	PLS win
	Fin_Be09	1.191	1.200	0.968	0.971	0.143	PLS win
	Fin_Be10	1.201	1.207	0.970	0.974	0.142	PLS win
	Fin_Be11	1.215	1.222	0.999	1.004	0.141	PLS win
	Fin_Be12	1.194	1.202	0.962	0.965	0.145	PLS win
Financial wellbeing	FWB_Scores	10.812	10.836	8.056	8.058	0.397	PLS win

Table 9A reported the PLSpredict results for unweighted model. All Q^2^_predict values (0.111–0.397) show that the model has meaningful predictive power but not noise. There are also lower RMSE and MAE in PLS than in LM, which indicates more accurate out-of-sample prediction.

RMSEs for Fin Behavior ranged between 1.192 and 1.234 (compared to LM’s); Q^2^_predict ranged from 0.111 to 0.150 (with the minimum from Fin_Be08 and the maximum from Fin_Be03; Table 9A). For Financial wellbeing, *Q*^2^_spredict for Financial wellbeing produced a positive *Q*^2^ (0.397) and significantly smaller prediction errors under PLS (RMSE = 10.810; MAE = 8.052) than LM. These are small differences in some cases and consistent with what we would expect from the structural model if the relationships are well described.

Following the recommendations of Hair et al. (2022) and Shmueli et al. (2019), the overall pattern suggests that the model is medium to strong predictive. The PLS-SEM, which is specifically suitable for higher-order constructs, gives steady performance for both indicators and latent ones. The weighted PLSpredict analysis also follows similar direction (Table 9B), validating the robustness of the model to predicting financial behavior and Financial wellbeing performance.

#### 4.6.2 Robustness and endogeneity tests

In order to further confirm the stability of the structural model and the possible endogeneity issues, additional tests were conducted to test another model that removed the mediator, investigated possible reverse causal relations and performed the Gaussian Copula procedure to detect possible end heterogeneity. All of these results are consistent with previous PLS-SEM results in evaluating the robustness and plausibility of the proposed causal ordering (Hair et al., 2022). Unless otherwise stated, robustness and endogeneity checks were conducted for both the unweighted and population-weighted models to ensure that conclusions are not driven by the weighting scheme. Where results are reported in parallel (e.g., reverse-causality models and Gaussian Copula tests), we provide side-by-side evidence to facilitate direct comparison across specifications.

##### 4.6.2.1 Alternative model checks

###### 4.6.2.1.1 Direct-only model

For testing if our proposed mediation structure actually predicts these associations, we used a direct-only model that does not do Financial Behavior. As noted in Table 10 Psychological Capital still has a good and significant positive effect in the unweighted model (*β* = 0.645, *p* < 0.001) and the weighted model (*β* = 0.721, *p* < 0.001). Clearly, the main link between the two cannot be explained with the mediator.

**Table 10:** Direct-only models under unweighted and weighted PLS-SEM.

Path	Model	*β*	*t*	*p*	Comment
Psychological capital - > Financial wellbeing	Unweighted	0.645	21.116	0.000	Stable and highly significant direct effect, indicating that the core relationship is not model-dependent
Psychological capital - > Financial wellbeing	Weighted	0.721	13.514	0.000	Direct effect remains positive and significant under weighting, confirming consistency of the relationship.

Although we see the behavioral path coefficient is smaller after weighting, this can be in fact an expected reduction in the effective sample size and more importantly, the direction and structure of relations remain stable. We conclude this helps us improve the character of the mechanism of hypothesized behavior and we address a question of the model-induced endogeneity.

###### 4.6.2.1.2 Reverse causality model

To probe whether reverse causality might explain the observed relationships, an alternative specification was estimated in which Financial wellbeing was modeled as predicting Financial Behavior. As reported in Table 11, this reversed pathway (Financial Wellbeing to Financial Behavior) reaches statistical significance only in the unweighted estimation and loses significance once population weights are applied. By contrast, the original paths from Psychological Capital to both Financial Behavior and Financial Wellbeing remain consistently strong and significant across weighted and unweighted models. Taken together, these patterns suggest that the proposed direction of influence (PsyCap to FWB via FB) is considerably more plausible, and any concerns regarding reverse causality appear limited.

**Table 11:** Robustness check via reverse path model.

Path	Model	*β*	*t*	*p*	Comment
Financial wellbeing - > Financial behavior	Unweighted	0.229	3.966	0.000	Weak but significant alternative influence; substantially smaller than theorized path
Psychological capital - > Financial behavior	Unweighted	0.322	5.847	0.000	Robust and significant, consistent with theoretical model
Psychological capital - > Financial WELLBEING	Unweighted	0.645	21.045	0.000	Strong and significant direct effect, confirming core model
Financial wellbeing - > Financial behavior	Weighted	0.144	1.386	0.166	Non-significant; reverse effect not supported when accounting for population weights
Psychological capital - > Financial behavior	Weighted	0.448	4.197	0.000	Effect remains significant; robust to weighting
Psychological capital - > Financial wellbeing	Weighted	0.713	13.431	0.000	Strong and significant direct effect; strengthened under weighting

In general, the other model tests indicate that the connection between psychological capital and financial wellbeing is stable and well supported in theory. When the mediator is removed, the direct-only model still shows a high and positive value of psychological capital with the population weights which indicates that this connection does not rely on a particular model configuration. At the same time, the reverse-causality checks show that the alternative path from psychological capital to financial behavior is weak and disappears after the weights have been removed. The hypotheses, however, hold up for specifications. Together, they enhance confidence in the causal path from psychology capital to financial behavior and finally financial well–being and remove model-driven artefacts or reciprocal causality (Hair et al., 2022).

##### 4.6.2.2 Gaussian copula endogeneity test

To assess whether endogeneity might be present among the latent constructs, this study applied the Gaussian Copula procedure (Sarstedt et al., 2019) to each structural path. The tests were run for the unweighted and weighted models separately, partly to confirm that incorporating population weights would not alter the underlying causal interpretation. As shown in Table 12, none of the Copula terms were statistically significant (all *p* > 0.05), indicating that endogeneity is unlikely to bias the parameter estimates.

**Table 12:** Gaussian copula endogeneity test results for unweighted and weighted models.

Model	Path	*β*	*t*	*p*
Unweighted	GC (Financial behavior) - > Financial wellbeing	0.074	0.819	0.413
	GC (Psychological capital) - > Financial behavior	0.257	1.946	0.052
	GC (Psychological capital) - > Financial wellbeing	0.208	1.351	0.177
Weighted	GC (Psychological capital) - > Financial behavior	0.053	0.262	0.793
	GC (Psychological capital) - > Financial wellbeing	0.116	0.407	0.684
	GC (Financial behavior) - > Financial wellbeing	0.138	0.899	0.369

GC = Gaussian Copula term; non-significant *p*-values (*p* > 0.05) indicate the absence of endogeneity bias. Bootstrap resamples = 10,000.

Specifically, in the unweighted model, the Copula terms for all examined relationships were not statistically significant. A similar pattern emerged in the weighted model, where the corresponding paths also failed to reach significance. The consistency of findings across both specifications demonstrates that the inclusion of population weights did not introduce or obscure potential endogeneity.

Overall, these results confirmed that the relationships among Psychological Capital, Financial Behavior, and Financial wellbeing are not driven by omitted variable bias or reciprocal causation.

##### 4.6.2.3 Summary of robustness

In short, the robustness analyses consistently validate the stability and causal credibility of the proposed structural model. The direct-only model confirmed that the core relationship between Psychological Capital and Financial Wellbeing remains strong and significant even without the mediator, indicating that the findings are not model-dependent. The reverse-causality model further showed that the alternative direction from Financial Wellbeing to Financial Behavior is weak and becomes non-significant after applying population weights, thereby supporting the theorized causal pathway (Psychological Capital → Financial Behavior → Financial Wellbeing). In addition, the Gaussian Copula tests produced non-significant outcomes for every structural path in both the unweighted and weighted models, suggesting that endogeneity is unlikely to distort the estimated relationships. Taken together, these findings indicate that the proposed associations are not only theoretically grounded but also empirically stable and largely unaffected by concerns related to endogeneity or model misspecification (Hair et al., 2022; Sarstedt et al., 2019). Building on this foundation, Section 4.7 presents the multi-group and invariance analyses, which offer a closer look at the consistency of these effects across key demographic groups.

#### 4.6.3 Weighting sensitivity analysis (extreme-weight trimming)

Given the dispersion of the sampling-weight distribution (Table 1C) and the substantial reduction in effective sample size under population weighting, we conducted a sensitivity analysis to examine whether the weighted structural estimates were unduly influenced by a small number of highly weighted observations. Specifically, we winsorized the sampling-weight variable at the 95th percentile (P95), such that weights above P95 were set to P95 while all other weights remained unchanged. We then re-estimated the population-weighted PLS-SEM model using the trimmed weights and repeated bootstrapping with 10,000 resamples. The results are summarized in Appendix Table 3, which presents a side-by-side comparison of the unweighted model, the original weighted model, and the weighted model with P95-trimmed weights.

Overall, the sensitivity analysis indicates that the overall distribution of results are consistent with extreme-weight trimming. The direct effects of Psychological Capital on Financial wellbeing and Financial Behavior remain positive and statistically significant for each model (unweighted: *β* = 0.563, *p* < 0.001; weighted: *β* = 0.657, *p* < 0.001; P95-trimmed: *β* = 0.669, *p* < 0.001; and unweighted: *β* = 0.469, *p* < 0.001; weighted: *β* = 0.551, *p* < 0.001; P95-trimmed: *β* = 0.532, p < 0.001; Appendix Table 3). Compared with the original model, the behavioral pathway from Financial Behavior to Financial wellbeing is not significant under population weighting, and our conclusion is unchanged after trimming (weighted: *β* = 0.103, *p* = 0.141; P95-trimmed: *β* = 0.095, *p* = 0.162). Importantly, the indirect effect of Psychological Capital on Financial wellbeing via Financial Behavior also remains positive and significant in the original weighted model and the P95-trimming model (weighted: *β* = 0.057, *p* = 0.154; P95-trimmed: *β* = 0.050, *p* = 0.179). These results suggest that the observed reduction of the behavioral pathway in the weighted model is not caused by a small number of extreme weights but rather due to combination of weighting-induced reductions of effective sample size and potential demographic heterogeneity of the weighting scheme.

Finally, Appendix Table 3 shows that the macroeconomic control paths (CPI, disposable income, and housing price index) remain small in magnitude and statistically non-significant after trimming, and the direction of these coefficients is largely stable across specifications. Taken together, the trimming-based sensitivity analysis strengthens confidence in the robustness of our key inferences: the direct effect of Psychological Capital on Financial Wellbeing is stable and generalizable under population weighting, whereas evidence for the behavioral mediation mechanism is more conservative in weighted estimations.

#### 4.6.4 Theoretical interpretation of findings through COR and SDT

Results can be interpreted from both Conservation of Resources (COR) perspectives and Self-Determination Theory (SDT) perspectives: From COR perspective, Psychological Capital is a source of personal resources (hope, self-efficacy, resilience, and optimism) that helps individuals protect from resource losses and invest resources to generate future gains under financial stress (Hobfoll, 2010; Hobfoll et al., 2018). Psychological Capital can be understood closely from COR standpoint: Individuals with richer psychological resources are more likely to perceive financial problems as manageable, feel confident to cope, and can feel more confident and secure for achieving higher financial wellbeing.

SDT also clarifies the motivation. Psychological Capital (i.e., hope and self-efficacy) can support basic psychological needs of competence and autonomy and promote more self-regulation and self-validated financial behavior (Ryan and Deci, 2017). The chain of Psychological Capital to Financial wellbeing via financial behavior in the unweighted model follows this SDT trend, suggesting that psychologically empowered individuals take budgeting, saving and planning for financial goals for psychological reasons.

Notably, the mediation pathway becomes more conservative under population weighting. Interpreted through COR and SDT, this attenuation suggests that the translation of psychological resources into behavioral gains may be more sensitive to population heterogeneity and contextual constraints: for some demographic segments, structural barriers and competing obligations may limit the extent to which autonomous motivation and perceived competence can be converted into consistent financial behaviors. Accordingly, COR primarily supports the robustness of the direct “resource-to-wellbeing” link, while SDT helps explain why the behavioral transmission mechanism may vary across population subgroups.

### 4.7 Multi-group and invariance analysis

In order to see if the structural relationship among major groups varies across individuals, we performed the Multi-Group Analyses (MGA). Before comparing path coefficients, we used the Measurement Invariance of Composite Models (MICOM) assessment to investigate both configural and compositional invariance (Henseler et al., 2016). Since MICOM indicated that we already had partial measurement invariance, which is generally sufficient, we proceeded by cross-group comparisons, which may be viewed as meaningful analytically. MICOM and subsequent multi-group comparisons were conducted using the unweighted dataset. This choice is motivated by two considerations. First, sampling weights were calibrated to match overall population margins (age, gender, education, and region) and are primarily intended to improve population representativeness in the full-sample structural estimates, whereas applying the same weights within subgroups can yield unstable estimates due to substantially reduced effective sample sizes. Income is reported in Table 1A for descriptive purposes only and was not used in the raking procedure; the sampling weights were calibrated using age, gender, education, and region as shown in Table 1B. Second, the purpose of MGA in this study is to examine heterogeneity of structural relationships across groups; therefore, we treat these analyses as exploratory robustness checks complementing the full-sample weighted/unweighted comparisons reported earlier (see Table 2).

#### 4.7.1 Measurement invariance assessment in MICOM

Before performing multi-group analysis, we followed the MGA step of Henseler et al. (2016) in order to ensure comparisons between demographic groups are meaningful but not affected by the measurement differences between group-based models.

##### 4.7.1.1 Step 1: configural invariance

Configural invariance was established for all group comparisons, as each subgroup model shared identical indicators, data treatment procedures, and algorithm settings in SmartPLS 4.1.0.3. This satisfies the baseline requirement for subsequent invariance testing.

##### 4.7.1.2 Step 2: compositional invariance

The permutation test results demonstrated that most latent constructs achieved high correlations (c ranging from 0.97 to 1.00) and nonsignificant permutation *p*-values (*p* > 0.05), confirming compositional invariance across gender, education, age, and regional groups (see Table 13 and Appendix Table 4–8). Minor deviations were observed in the *Financial Wellbeing* construct among age groups and in the *Financial Behavior* construct among regional groups, where a few permutation p-values fell slightly below 0.05. However, these deviations were limited to control or secondary variables, and did not affect the measurement equivalence of the main theoretical constructs (*Psychological Capital*, *Financial Behavior*, and *Financial Wellbeing*).

**Table 13:** Summary of MICOM results across demographic groups.

Grouping variable	No. of groups	Configural invariance	Compositional invariance (step 2)	Equality of means/variances (step 3)	Decision
Gender	2	Established	Supported (*p* > 0.05 for all constructs)	Supported (*p* > 0.05)	Full invariance
Education	2	Established	Supported (*p* > 0.05 for all constructs)	Supported (*p* > 0.05)	Full invariance
Age	4	Established	Largely supported (minor *p* < 0.05 for FWB)	Partial (minor variance differences)	Partial invariance
Region	4	Established	Mixed (several *p* < 0.05 for FB and FWB)	Partial (*p* > 0.05 for most constructs)	Partial invariance

##### 4.7.1.3 Step 3: equality of means and variances

Permutation tests further indicated no systematic differences in means or variances for the majority of constructs (*p* > 0.05), supporting at least partial measurement invariance. Specifically, all constructs demonstrated mean and variance equality across gender and education groups, while only minor variations appeared across age and regional groups.

As summarized in Table 13, full measurement invariance was achieved for gender and education groups, while partial invariance was established for age and regional groups. According to Hair et al. (2022) and Henseler et al. (2016), partial invariance is sufficient for comparing structural relationships across groups in the subsequent MGA. Therefore, the study proceeded to multi-group analysis to explore potential differences in structural path coefficients across demographic subpopulations.

#### 4.7.2 Multi-group analyses results

To examine whether the structural relationships differ across demographic subgroups, a series of MGA were conducted using the nonparametric PLS-MGA approach with 10,000 bootstrapping resamples (Hair et al., 2022). This procedure compares group-specific path coefficients and evaluates the probability that their difference is significantly different from zero. A difference is considered statistically significant when *p* < 0.05 or *p* > 0.95.

As shown in Appendix Table 8, cross gender, education, and age groups, all path differences were nonsignificant (*p* > 0.05), suggesting that the effects of Psychological Capital on Financial Behavior and Financial wellbeing are stable across these demographic categories. The behavioral mediation mechanism (Psychological Capital → Financial Behavior → Financial Wellbeing) therefore appears generally consistent across gender, education, and age groups in this sample.

However, several regional comparisons showed larger differences in coefficient. For example, for both the Center and North East regions all three structural paths showed significant differences (*p* < 0.001). Although this result should be seen with caution, only 56 persons are in the North East subgroup, which is below the recommended sample size for PLS-SEM subgroup estimation. The path estimates might also be unstable and may overstate apparent differences. Importantly, the regional MGA results should be treated as exploratory rather than confirmatory. The small North-East subgroup (*n* = 56) implies limited statistical power and potentially unstable bootstrap estimates, and the multiple pairwise regional comparisons increase the risk of chance findings. Therefore, we avoid strong claims about region-specific mechanisms and interpret apparent regional differences as preliminary signals that warrant replication in larger, more balanced regional samples (or probability-based datasets) before substantive conclusions are drawn.

Other regional contrasts (Center vs. East, East vs. West, etc.) have mixed results: While the psychological capital vs. financial wellbeing path difference in some comparisons (East vs. West *p* = 0.002), most other relationships are consistent. Taken together, the MGA results imply that the proposed model is highly invariant in gender, education, and age groups, and the relationships are stable between regions, only slightly regional variation from group sizes are likely.

#### 4.7.3 Discussion of group differences

Our multi-group results suggest that our proposed structural relations are in general stable in demographic groups, suggesting that psychological capital – financial behavior – financial wellbeing mechanism is broadly applicable, respectively. For example, the general pattern of gender, education, and age groups suggests that psychological capital’s positive impact on financial behavior and financial wellbeing is a general psychological process, and not a group-based process. This fits the idea that psychological resources like hope, resilience, effectiveness and optimism cross-situational drivers of adaptive behavior and subjective wellbeing (Luthans et al., 2007b; Youssef-Morgan and Luthans, 2015).

The lack of significant differences between most individuals suggests that these differences do not help significantly constrain the structural relationship of the model, suggesting that psychological capital is, rather, a context independent internal resource (Sweetman et al., 2011). Still, the slight variations that emerged in regional comparisons, especially between the Center and North East regions, may stem more from socioeconomic disparities or uneven financial infrastructure than from substantive psychological differences (Brüggen et al., 2017). As the North East subgroup had a small sample size (*n* = 56), its apparent significance should be interpreted cautiously due to limited statistical power.

Overall, these findings suggest that Psychological Capital–based approaches may be broadly relevant for improving financial behavior and financial wellbeing in the Chinese context. However, any region-specific inferences should be interpreted cautiously and tested in future studies with larger and more balanced regional samples.

### 4.8 Model fit assessment

To evaluate the global goodness-of-fit of the structural model, two widely accepted indices in PLS-SEM were examined based on the Standardized Root Mean Square Residual (SRMR) and the Normed Fit Index (NFI). As shown in Table 14, the composite-based model yielded SRMR = 0.028 and NFI = 0.973, both within the recommended thresholds (SRMR < 0.08 from Henseler et al. (2015); NFI > 0.90 from Hair et al. (2022)).

**Table 14:** Model fit from PLS algorithm of second stage.

Fit indices	Estimated model
SRMR	0.028
NFI	0.973

These results indicate satisfactory model adequacy and suggest that the structural model exhibits minimal misspecification. It is important to note, however, that in PLS-SEM, such indices serve as approximate rather than absolute indicators of fit, given the component-based nature of the estimation procedure (Henseler et al., 2015). While traditional CB-SEM reports global fit indices such as CFI or RMSEA, PLS-SEM reports SRMR as a standard indicator of residuals to ensure that the model does match data. Here, the high NFI value adds additional reassurance about the model’s explanatory strength and sufficientness.

## 5 Conclusion and implications

The main statistical results of the mediation model (psychological capital → Financial behavior → Financial wellbeing) applied to the unweighted and population weighted PLS-SEM are as follows. First, the positive and significant effect of Psychical Capital on Financial wellbeing was strong and significant for both models (unweighted: *β* = 0.563, *p* < 0.001; weighted: *β* = 0.657, *p* < 0.001; Tables 2A,B) with respect to Hypothesis 1. Second, the impacts from Psychological Capital on Financial Behavior (unweighted: *β* = 0.469, *p* < 0.001; weighted: *β* = 0.551, *p* < 0.001; Tables 2A,B) were strong and meaningful in both models. Third, the effect of Financial Behavior on Financial wellbeing was positive and significant in the unweighted model (*β* = 0.174, *p* < 0.001; Table 2A) but statistically non-significant in the weighted model (*β* = 0.103, *p* = 0.141; Table 2B). Meanwhile, the results led a significant partial mediation effect in the unweighted model (*β* = 0.081, *p* < 0.001; Table 2A, supporting H2) and a non-significant mediation effect in the weighted model (*β* = 0.057, *p* = 0.154; Table 2B), which indicate the mediation mechanism is sensitive to demographic heterogeneity. Finally, provincial macroeconomic control variables (CPI, disposable income, housing price index) have no major effect on Financial Behavior or Financial Wellbeing in either model, suggesting the psychological-behavior mechanism is independent of local economic conditions.

Building on these core statistical findings, we investigated how psychological capital relates to financial wellbeing by examining the mediating role of financial behavior among working-age adults in China. In doing so, we provide additional theoretical support by modeling psychological capital as a formative higher-order construct and by assessing the robustness of the focal relationships using both unweighted and population-weighted PLS-SEM estimations, complemented by predictive diagnostics and endogeneity checks—underscoring the robustness and external validity of the core direct effect.

Psychological Capital positively predicted Financial wellbeing (*β* = 0.563, *p* < 0.001; Table 2A), supporting Hypothesis 1. Psychological Capital also positively predicted Financial Behavior (*β* = 0.469, *p* < 0.001; Table 2A), and Financial Behavior positively predicted Financial Wellbeing (*β* = 0.174, *p* < 0.001; Table 2A), with a significant indirect effect (*β* = 0.081, *p* < 0.001; Table 2A) supporting partial mediation (Hypothesis 2). When probability weights were applied… the behavioral pathway (*β* = 0.103, *p* = 0.141; Table 2B) and indirect effect (*β* = 0.057, *p* = 0.154; Table 2B) were statistically non-significant. Our results suggest that our strongest and most general result is that psychological capital positively predicts financial wellbeing and that the mediation mechanism via financial behavior should be interpreted more cautiously as sensitive to demographic heterogeneity and low effective sample under weights. These findings are consistent with Conservation of Resources theory (Hobfoll, 2010; Hobfoll et al., 2018) which argues psychological resources (hope, resilience, optimism, self-efficacy) facilitate adaptive behaviors that financial wellbeing. Our model thus extends financial wellbeing literature by highlighting psychological strengths as a strong predictor of financial outcomes, and in indicating behavior-based mechanism, which may be more conservative if population representation is imposed.

This article contributes to the discussion of Financial Wellbeing by bringing psychological capital theory into perspective with the behavioral models in Brüggen et al. (2017) model. In contrast to economic explanations, we find that psychological resources (particularly hope, resilience, optimism, and self-efficacy) help the individuals make better decisions and also the social wellbeing. By modeling psychological capital as a second-order formative model, and empirically testing its effects on a behavioral pathway, we add interest to a growing literature that treats psychological traits as adjustable assets for personal financial decisions (Goyal et al., 2021; Luthans and Youssef-Morgan, 2017). Our results further extend Conservation of Resources theory with the study that psychological capital may be a reservoir that motivates the individuals to perform more proactive financial behaviors (Hobfoll, 2010). At the same time, the mediated model is compatible with Self-Determination Theory, suggesting that financial behavior can be regarded as an expression of self-interested motivation driven by internal psychology (Deci and Ryan, 2000; Ryan and Deci, 2017) and, together, offer a deeper psychological understanding of Financial Wellbeing beyond structural or material factors.

At the component level, the formative outer-weight results (Appendix Table 1) suggest that resilience gives the highest order Psychological Capital structure in both the unweighted and weighted results, suggesting adaptive coping may be much more important to keep perceived financial security in the Chinese case. Self-efficacy seems to suffer at population weighting, suggesting competence-related resources could be different across demography, and may be more sensitive to population heterogeneity when representativeness is imposed.

These results suggest possible ways to improve financial wellbeing of working-age adults in China. First, financial education activities may be designed as “dual-component” programs that use skills training and psychological capital development, (i.e., hope building modules may guide people to turn long-term goals, such as housing, education, retirement) into feasible subgoals and alternative strategies; (b) self-efficacy coaching can guide people into budgeting, bill management, and debt repayment plans with increasing difficulty, and resilience-related modules can stress coping plans for income shocks such as emergency-funds routines and scenario planning, and (d) realistic optimism modules can encourage forward thinking but not fear of risky investments. Second, employers and community organizations (e., union, neighborhood committee, community service centers) may embed short psychological capital micro-interventions (e.g., 2–4-week modules) into workplace wellness/community support programs to target subgroups with greater financial responsibilities and stress (e.g., middle-aged households and lower-education groups highlighted by the population-weighting diagnostics). Third, banks and fintech platforms can incorporate behavioral prompts and supportive feedback (e.g., saving-plan reminders, progress dashboards and default options for automatic transfers) to promote autonomy and competence, which align with SDT methods to sustain responsible financial behavior over time. Program evaluations can use changes in core financial behaviors (budgeting, saving, debt management and insurance planning) as proximal indicators, and perceived financial health is monitored as downstream outcome.

Since psychological capital is a state of the art, and it can be used in a permanent way (Luthans et al., 2007b), it provides a concrete platform for programs aimed at improving long-term financial wellbeing. Financial education projects could become more successful if they incorporate psychological capital development through hope building and efficacy coaching, and resilience training as a part of traditional financial literacy content. Together, cognitive and motivating aspects could provide sustainable improvements to financial wellbeing. Meanwhile, China’s institutional structure, characterized by rapid digital financial developments and government-driven “common prosperity” reforms, makes room for interventions that operate on both the psychological and behavioral domains. Policy makers, educators, and financial institutions may also consider designing programs that not only transfer financial knowledge, but also empower people to act on it, using this kind of programs. These approaches are more appropriate in order to address socioeconomic differences and long-lasting financial resiliency.

These results should be interpreted as China’s cultural and institutional situation, in which collectivist expectations and intergenerational burdens make financial decisions. Many middle-aged adults face the so-called “sandwich generation,” carrying out housing payments, school education and eldercare tasks at the same time. These demands not only stress financial resources, but also lead to persistent psychological stresses and less control over future financial outcomes. Due to the lack of research on psychological capital in China, it is hard to design strategies which improve financial stability and wellbeing (Khan et al., 2024). It is crucial to address this issue as knowledge how hope, resilience and other psychological resources help individuals to cope with intergenerative burdens can inform decisions to improve financial health across the globe.

The digital financial services such as Alipay and WeChat Pay are increasingly gaining popularity among young users. While these technologies can provide financial access to urban users, they can contribute to impulsive spending and high debt risk for older or poorer people in rural regions. This raises the question: how to combine psychological capital to deal with cognitive and behavioral issues in financial decision making, where policy decisions should go beyond traditional literacy. Incorporating psychological capital development in education and policy frameworks to empower people to better control their future financial lives. Further systemic limitations in China’s financial world will eventually emerge through multi-level efforts that combine psychological resources with practical financial resources. In this regard, the “Psychological Capital + Behavior” framework proposed here can be developed as a theoretical and practical basis to support financial resilience and reduce socioeconomic inequalities, especially during growing economic uncertainty in developing countries such as China.

## 6 Limitations and recommendations

Although our work provides a better understanding of psychological and behavioral aspects of financial wellbeing for adults in China, it has several caveats. Although subgroup analyses were carried out for gender, education, age, and region, it should be possible to work more collaboratively with larger and more evenly distributed samples as more and more balanced data would benefit both statistical power and internal validity. More demographic and geographic diversity, which is only partly addressed here, may also allow more reliable subgroup comparisons. Moreover, the cross-sectional nature of our design limits causal interpretations. Even though our structural model is highly robust, longitudinal or experimental studies would be better suited to analyze causal processes and investigate if particular intervention to improve psychological capital can improve financial behavior and financial wellbeing.

Although Financial Behavior was a useful input in this study, future work may include other forms of expression such as emotion control, work activity, or subjective financial knowledge to further understand how psychological resources interact with financial actions and outcomes. Such additional measures may reveal pathways which are beyond the scope of the current design. Finally, cross-cultural or international comparisons may be necessary to investigate the applicability of the proposed model because cultural expectations, institutions and policy settings will always determine how psychological traits contribute to financial wellbeing.

## Data Availability

The original contributions presented in the study are included in the article/Supplementary material, further inquiries can be directed to the corresponding author.

## Footnotes

1
https://www.wjx.cn

2
https://www.stats.gov.cn
