# Supplementary material for: The mediating role of financial behavior in the relationship between psychological capital and financial wellbeing: evidence from a cross-sectional study in China
Source: Front Psychol. 2026 Apr 14;17:1760291. doi: 10.3389/fpsyg.2026.1760291 (PMC13121066; doi:10.3389/fpsyg.2026.1760291)
Supplement: Supplementary file 1 [file Table_1.DOCX]

**Appendix Table 1. Formative Outer Weights for PsyCap in Second Stage (Unweighted and Weighted)**

| Indicator | Unweighted Model | | | | | Weighted Model | | | | |
| --- | --- | --- | --- | --- | --- | --- | --- | --- | --- | --- |
|  | *β* | *p* | CI | | VIF | *β* | *p* | CI | | VIF |
|  |  |  | 2.5% | 97.5% |  |  |  | 2.5% | 97.5% |  |
| LV scores - Hope -> Psychological Capital | 0.350 | 0.000 | 0.216 | 0.477 | 1.252 | 0.325 | 0.000 | 0.141 | 0.506 | 1.326 |
| LV scores - Optimism -> Psychological Capital | 0.312 | 0.000 | 0.189 | 0.431 | 1.277 | 0.394 | 0.000 | 0.188 | 0.598 | 1.286 |
| LV scores - Resilience -> Psychological Capital | 0.390 | 0.000 | 0.255 | 0.522 | 1.494 | 0.429 | 0.000 | 0.222 | 0.615 | 1.711 |
| LV scores - Self-Efficacy -> Psychological Capital | 0.326 | 0.000 | 0.192 | 0.450 | 1.319 | 0.181 | 0.056 | 0.010 | 0.383 | 1.353 |

**Appendix Table 2. Outer Loadings for All Indicators (Unweighted and Weighted)**

| Indicators | Outer Loadings in Unweighted Model | Outer Loadings in Weighted Model |
| --- | --- | --- |
| Fin_Be01 <- Financial Behavior | 0.798 | 0.794 |
| Fin_Be02 <- Financial Behavior | 0.778 | 0.819 |
| Fin_Be03 <- Financial Behavior | 0.807 | 0.810 |
| Fin_Be04 <- Financial Behavior | 0.815 | 0.809 |
| Fin_Be05 <- Financial Behavior | 0.808 | 0.834 |
| Fin_Be06 <- Financial Behavior | 0.786 | 0.782 |
| Fin_Be07 <- Financial Behavior | 0.798 | 0.818 |
| Fin_Be08 <- Financial Behavior | 0.809 | 0.805 |
| Fin_Be09 <- Financial Behavior | 0.809 | 0.810 |
| Fin_Be10 <- Financial Behavior | 0.821 | 0.799 |
| Fin_Be11 <- Financial Behavior | 0.808 | 0.769 |
| Fin_Be12 <- Financial Behavior | 0.803 | 0.802 |
| Hope01 <- Hope | 0.782 | 0.747 |
| Hope02 <- Hope | 0.805 | 0.813 |
| Hope03 <- Hope | 0.762 | 0.789 |
| Hope04 <- Hope | 0.772 | 0.780 |
| Hope05 <- Hope | 0.783 | 0.793 |
| Hope06 <- Hope | 0.787 | 0.747 |
| Opt01 <- Optimism | 0.799 | 0.789 |
| Opt02 <- Optimism | 0.800 | 0.802 |
| Opt03 <- Optimism | 0.774 | 0.861 |
| Opt04 <- Optimism | 0.774 | 0.774 |
| Opt05 <- Optimism | 0.767 | 0.814 |
| Opt06 <- Optimism | 0.774 | 0.820 |
| Opt07 <- Optimism | 0.792 | 0.757 |
| Opt08 <- Optimism | 0.791 | 0.814 |
| Res01 <- Resilience | 0.778 | 0.774 |
| Res02 <- Resilience | 0.813 | 0.833 |
| Res03 <- Resilience | 0.789 | 0.764 |
| Res04 <- Resilience | 0.784 | 0.764 |
| Res05 <- Resilience | 0.813 | 0.779 |
| Res06 <- Resilience | 0.826 | 0.867 |
| Res07 <- Resilience | 0.784 | 0.773 |
| Res08 <- Resilience | 0.805 | 0.817 |
| Res09 <- Resilience | 0.796 | 0.778 |
| Res10 <- Resilience | 0.784 | 0.801 |
| Self_Eff01 <- Self-Efficacy | 0.808 | 0.830 |
| Self_Eff02 <- Self-Efficacy | 0.829 | 0.836 |
| Self_Eff03 <- Self-Efficacy | 0.803 | 0.792 |
| Self_Eff04 <- Self-Efficacy | 0.805 | 0.840 |
| Self_Eff05 <- Self-Efficacy | 0.792 | 0.764 |
| Self_Eff06 <- Self-Efficacy | 0.814 | 0.817 |
| Self_Eff07 <- Self-Efficacy | 0.801 | 0.817 |
| Self_Eff08 <- Self-Efficacy | 0.763 | 0.759 |
| Self_Eff09 <- Self-Efficacy | 0.828 | 0.848 |
| Self_Eff10 <- Self-Efficacy | 0.816 | 0.812 |

**Appendix Table 3. Sensitivity analysis of key paths under extreme-weight trimming (P95 winsorization)**

| Type | Path | Unweighted Model | | Weighted Model | | Weighted Model (P95-trimmed) | |
| --- | --- | --- | --- | --- | --- | --- | --- |
|  |  | *β* | *p* | *β* | *p* | *β* | *p* |
| Direct | Psychological Capital -> Financial Well-Being | 0.563 | 0.000 | 0.657 | 0.000 | 0.669 | 0.000 |
|  | Psychological Capital -> Financial Behavior | 0.469 | 0.000 | 0.551 | 0.000 | 0.532 | 0.000 |
|  | Financial Behavior -> Financial Well-Being | 0.174 | 0.000 | 0.103 | 0.141 | 0.095 | 0.162 |
| Indirect | Psychological Capital -> Financial Behavior -> Financial Well-Being | 0.081 | 0.000 | 0.057 | 0.154 | 0.050 | 0.179 |
| Control Variables | CPI-> Financial Behavior | 0.023 | 0.600 | 0.104 | 0.134 | 0.088 | 0.202 |
|  | CPI -> Financial Well-Being | 0.020 | 0.580 | 0.076 | 0.149 | 0.077 | 0.109 |
|  | Disposable Income -> Financial Behavior | -0.048 | 0.299 | -0.084 | 0.263 | -0.078 | 0.305 |
|  | Disposable Income -> Financial Well-Being | 0.050 | 0.201 | 0.100 | 0.130 | 0.075 | 0.199 |
|  | Housing Price Index -> Financial Behavior | -0.023 | 0.591 | 0.024 | 0.690 | 0.027 | 0.669 |
|  | Housing Price Index -> Financial Well-Being | -0.022 | 0.530 | -0.084 | 0.180 | -0.039 | 0.444 |

**Appendix Table 4. MICOM Results for Gender Groups (2 groups)**

| Construct | Correlation (c) | p (compositional) | Mean Diff p | Variance Diff p |
| --- | --- | --- | --- | --- |
| Psychological Capital | 0.976 | 0.415 | 0.662 | 0.599 |
| Financial Behavior | 0.999 | 0.242 | 0.505 | 0.618 |
| Financial Well-Being | 1.000 | 0.353 | 0.389 | 0.228 |

**Appendix Table 5. MICOM Results for Education Groups (2 groups)**

| Construct | Correlation (c) | p (compositional) | Mean Diff p | Variance Diff p |
| --- | --- | --- | --- | --- |
| Psychological Capital | 0.979 | 0.518 | 0.874 | 0.274 |
| Financial Behavior | 1.000 | 0.841 | 0.285 | 0.751 |
| Financial Well-Being | 1.000 | 0.422 | 0.597 | 0.586 |

**Appendix Table 6. MICOM Results for Age Groups (4 groups)**

| Construct | Correlation (c) | p (compositional) | Mean Diff p | Variance Diff p |
| --- | --- | --- | --- | --- |
| Psychological Capital | 0.973  0.977  0.967  0.978  0.991  0.993 | 0.590  0.679  0.706  0.589  0.935  0.960 | 0.427  0.674  0.181  0.134  0.434  0.055 | 0.119  0.481  0.161  0.244  0.120  0.296 |
| Financial Behavior | 0.999  0.999  0.998  0.999  0.998  0.999 | 0.267  0.286  0.089  0.235  0.214  0.366 | 0.857  0.605  0.300  0.681  0.316  0.495 | 0.800  0.368  0.104  0.422  0.385  0.366 |
| Financial Well-Being | 1.000  1.000  1.000  1.000  1.000  1.000 | 0.000  0.324  0.000  0.221  0.457  0.020 | 0.501  0.821  0.188  0.614  0.356  0.216 | 0.135  0.446  0.062  0.365  0.912  0.125 |

**Appendix Table 7. MICOM Results for Region Groups (4 groups)**

| Construct | Correlation (c) | p (compositional) | Mean Diff p | Variance Diff p |
| --- | --- | --- | --- | --- |
| Psychological Capital | 0.989  0.941  0.875  0.980  0.936  0.980 | 0.812  0.590  0.161  0.822  0.313  0.891 | 0.543  0.561  0.863  0.250  0.773  0.542 | 0.580  0.576  0.849  0.292  0.801  0.541 |
| Financial Behavior | 0.999  0.999  0.996  0.999  0.998  0.998 | 0.014  0.844  0.121  0.390  0.020  0.560 | 0.176  0.445  0.533  0.075  0.648  0.259 | 0.153  0.400  0.817  0.041  0.413  0.355 |
| Financial Well-Being | 1.000  1.000  1.000  1.000  1.000  1.000 | 0.382  0.000  0.440  0.000  0.551  0.000 | 0.992  0.886  0.801  0.873  0.783  0.936 | 0.527  0.747  0.820  0.877  0.792  0.922 |

**Appendix Table 8. MGA Results**

| Comparison | Path | β (Group 1) | β (Group 2) | Δβ | p-value (MGA) |
| --- | --- | --- | --- | --- | --- |
| Gender  (Male vs Female) | Psychological Capital → Financial Behavior | 0.465 | 0.485 | -0.020 | 0.822 |
|  | Financial Behavior → Financial Well-Being | 0.171 | 0.175 | -0.004 | 0.971 |
|  | Psychological Capital → Financial Well-Being | 0.574 | 0.559 | 0.015 | 0.852 |
| Education  (High vs Low) | Psychological Capital → Financial Behavior | 0.450 | 0.552 | -0.102 | 0.248 |
|  | Financial Behavior → Financial Well-Being | 0.189 | 0.105 | 0.084 | 0.407 |
|  | Psychological Capital → Financial Well-Being | 0.532 | 0.671 | -0.139 | 0.112 |
| Age  (20-29  vs  30-39) | Psychological Capital → Financial Behavior | 0.540 | 0.455 | 0.085 | 0.392 |
|  | Financial Behavior → Financial Well-Being | 0.272 | 0.071 | 0.201 | 0.118 |
|  | Psychological Capital → Financial Well-Being | 0.440 | 0.665 | -0.226 | 0.072 |
| Age  (20-29  vs  40-49) | Psychological Capital → Financial Behavior | 0.540 | 0.445 | 0.085 | 0.398 |
|  | Financial Behavior → Financial Well-Being | 0.272 | 0.196 | 0.076 | 0.577 |
|  | Psychological Capital → Financial Well-Being | 0.440 | 0.546 | -0.106 | 0.437 |
| Age  (20-29  vs  50-59) | Psychological Capital → Financial Behavior | 0.540 | 0.501 | -0.039 | 0.791 |
|  | Financial Behavior → Financial Well-Being | 0.272 | 0.297 | -0.025 | 0.882 |
|  | Psychological Capital → Financial Well-Being | 0.440 | 0.545 | -0.106 | 0.537 |
| Age  (30-39  vs  40-49) | Psychological Capital → Financial Behavior | 0.455 | 0.455 | 0.001 | 0.996 |
|  | Financial Behavior → Financial Well-Being | 0.071 | 0.196 | 0.120 | 0.233 |
|  | Psychological Capital → Financial Well-Being | 0.665 | 0.546 | -0.125 | 0.171 |
| Age  (30-39  vs  50-59) | Psychological Capital → Financial Behavior | 0.455 | 0.501 | -0.046 | 0.698 |
|  | Financial Behavior → Financial Well-Being | 0.071 | 0.297 | -0.227 | 0.134 |
|  | Psychological Capital → Financial Well-Being | 0.665 | 0.545 | 0.120 | 0.387 |
| Age  (40-49  vs  50-59) | Psychological Capital → Financial Behavior | 0.455 | 0.501 | -0.046 | 0.699 |
|  | Financial Behavior → Financial Well-Being | 0.196 | 0.297 | -0.102 | 0.517 |
|  | Psychological Capital → Financial Well-Being | 0.546 | 0.545 | 0.000 | 0.991 |
| Region  (Center  Vs  East) | Psychological Capital → Financial Behavior | 0.315 | 0.529 | -0.214 | 0.034 |
|  | Financial Behavior → Financial Well-Being | 0.163 | 0.255 | -0.092 | 0.374 |
|  | Psychological Capital → Financial Well-Being | 0.543 | 0.520 | 0.023 | 0.790 |
| Region  (Center  Vs  North East) | Psychological Capital → Financial Behavior | 0.315 | 0.486 | -0.171 | 0.000 |
|  | Financial Behavior → Financial Well-Being | 0.163 | 0.137 | 0.026 | 0.000 |
|  | Psychological Capital → Financial Well-Being | 0.543 | 0.520 | 0.022 | 0.000 |
| Region  (Center  Vs  West) | Psychological Capital → Financial Behavior | 0.315 | 0.434 | -0.119 | 0.435 |
|  | Financial Behavior → Financial Well-Being | 0.163 | -0.049 | 0.212 | 0.083 |
|  | Psychological Capital → Financial Well-Being | 0.543 | 0.822 | -0.279 | 0.006 |
| Region  (East  Vs  North  East) | Psychological Capital → Financial Behavior | 0.529 | 0.486 | 0.043 | 0.000 |
|  | Financial Behavior → Financial Well-Being | 0.255 | 0.137 | 0.118 | 0.000 |
|  | Psychological Capital → Financial Well-Being | 0.520 | 0.520 | 0.000 | 0.000 |
| Region  (East  Vs  West) | Psychological Capital → Financial Behavior | 0.529 | 0.434 | 0.095 | 0.482 |
|  | Financial Behavior → Financial Well-Being | 0.255 | -0.049 | 0.304 | 0.006 |
|  | Psychological Capital → Financial Well-Being | 0.520 | 0.822 | -0.302 | 0.002 |
| Region  (North East  Vs  West) | Psychological Capital → Financial Behavior | 0.486 | 0.434 | 0.052 | 0.000 |
|  | Financial Behavior → Financial Well-Being | 0.137 | -0.049 | 0.186 | 0.000 |
|  | Psychological Capital → Financial Well-Being | 0.520 | 0.822 | -0.302 | 0.000 |
